# Fully Oxidized State of the Oxygen-Tolerant [NiFe] Hydrogenase from *Hydrogenophilus thermoluteolus* SH: A Quantum Mechanics Cluster and Quantum Mechanics/Molecular Mechanics Study

**DOI:** 10.1021/acs.inorgchem.5c00503

**Published:** 2025-05-07

**Authors:** Ravi Kumar, Andrés M. Escorcia, Matthias Stein

**Affiliations:** Molecular Simulations and Design Group, Max Planck Institute for Dynamics of Complex Technical Systems, Sandtorstrasse 1, 39106 Magdeburg, Germany

## Abstract

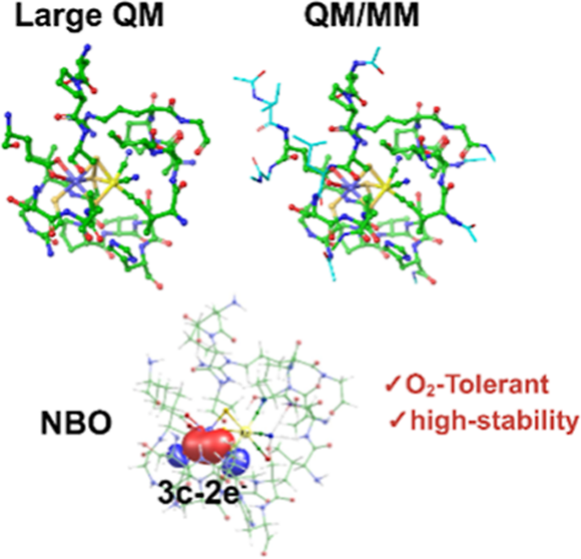

The oxygen tolerance of some [NiFe] hydrogenase enzymes is crucial for designing efficient bioinspired catalysts for sustainable hydrogen production and advancing renewable energy technologies. To investigate this, we employed a quantum mechanical (QM) cluster model and quantum mechanics/molecular mechanics (QM/MM) calculations to study the fully oxidized state of the [NiFe]-hydrogenase from *Hydrogenophilus thermoluteolus* SH. Our analysis focused on the structural and electronic properties of the enzyme’s active site across different spin states, including closed-shell singlet (CS, S = 0), high-spin triplet (HS, S = 1), and open-shell singlet broken symmetry (BS, S = 0). Using a comprehensive structural model (>300 atoms), we identified the ground state of the fully oxidized enzyme state to be a spin-coupled BS Ni(III)Fe(III) oxidation state, where residues beyond the first coordination sphere primarily contribute sterically. Notably, natural bond order calculations revealed an unusual three-center two-electron bond at the active site, which may enhance the open-shell ground state stability and the enzyme’s resilience under oxidative conditions. Our comparative study of QM and QM/MM methods provides insights into their performance, facilitating and guiding the choice of suitable enzyme models when studying other metalloproteins.

## Abstract 2

The oxygen tolerance of some [NiFe]-hydrogenase enzymes is crucial for designing efficient bioinspired catalysts for sustainable hydrogen production and advancing renewable energy technologies.

## Introduction

Molecular hydrogen (H_2_) is a next generation energy carrier accompanying the transition to sustainable power and achieving the 1.5 °C limit in accordance with the Paris scenario and a zero-emission strategy by 2050.^1−5^ Therefore, research focused on H_2_ production and utilization using hydrogenase enzymes has gained significant relevance in recent years.^6,7^ The active site of [NiFe] hydrogenase enzymes, comprising nickel and iron, two nonprecious and earth-abundant first–row transition metals, catalyzes the reversible proton reduction to H_2_.^8,9^

Their catalytic efficiency is comparable to or even surpasses that of platinum and other noble metal-based catalysts.^10−12^ Due to their catalytic power and selectivity, they are considered promising biocatalysts for the hydrogen economy.^6,7^ Hydrogenases can be used for a variety of applications, ranging from fuel cells to electro- and photocatalysis.^13−15^ However, most hydrogenases are sensitive to the presence of oxygen (O_2_), which limits their industrial application.^7,16,17^ Understanding the mechanisms behind oxygen tolerance in some hydrogenases is crucial, as it can help in the design of enzyme variants and bioinspired artificial systems with improved stability.^18−23^ Oxygen tolerance in hydrogenases has been linked to the presence of selenocysteines,^24^ narrow gas access channels to the active site,^25^ and an unusual proximal iron–sulfur cluster that protects the active site from oxidative damage.^26−28^

Recently, the oxygen tolerance of the [NiFe]-hydrogenase from the soluble NAD^+^-reducing *Hydrogenophilus thermoluteolus* (*Ht* SH) has been attributed to significant structural differences between the oxidized and reduced states.^29,30^ X-ray crystallography revealed an unusual coordination sphere at the nickel atom in the fully oxidized state of the enzyme, where a terminal cysteine residue (Cys462) is displaced by a bidentate coordinating Glu32 residue.^30^ Cys462 then occupies the position of a third μ-bridging cysteine. Based on minimal model calculations, IR and EPR spectral features were assigned to an unprecedented high-valent nickel Ni(IV) oxidation state in biological systems, resulting in an overall closed-shell Ni(IV)Fe(II) active site.^29^ We were able to show, however, that even for the minimal active site model, DFT calculations performed with a range of functionals (GGA BP86, hybrid functionals B3LYP, PBE0, and TPSSh) are fully consistent and they all give an energetically lower broken-symmetry Ni(III)Fe(III) state. This state is characterized by ligand-mediated antiferromagnetic spin-coupling between the metals, leading to an overall open-shell singlet (S = 0) spin state with evenly distributed spin densities over both metal atoms. In “standard” [NiFe] hydrogenase enzymes, only the nickel atom is redox active as it shuttles between Ni(III) EPR-active and Ni(II) EPR-silent oxidation states, with Ni(I) in the light-induced state being an exception. The iron remains in an EPR-silent low-spin Fe(II) (S = 0) state and was only later assigned by Mössbauer experiments. Thus, both the suggested superoxidation of nickel to a Ni(IV) or the oxidation of iron to an Fe(III) remain puzzling.

A realistic representation of enzyme active sites in quantum mechanical (QM) calculations is a complex issue, with various perspectives and aspects to consider.^31−36^ Small QM clusters offer computational efficiency, but they may lead to significant distortions of the active site and misinterpretations of the electronic properties. Considering the active site surroundings is essential for systems where long–range interactions play a crucial role in structural variation and site reactivity. Large QM cluster models, which include protein residues beyond the first coordination sphere, provide a more accurate and comprehensive understanding of enzyme catalytic properties. However, they are computationally expensive and more complex.^37−44^

Hybrid quantum mechanics/molecular mechanics (QM/MM) calculations enable consideration of the active site surroundings by splitting the enzyme into regions of QM (computationally expensive) and MM (computationally less demanding) levels of theory.^34−36,45^ In QM/MM calculations, the QM region typically comprises the active site, while the surrounding environment is treated by using MM methods. QM/MM calculations are generally less computationally demanding than large QM cluster calculations. The accuracy of QM/MM calculations can depend on the size of the QM region, which may need to be extended to include a large portion of the active site surroundings. In such cases, the computational demand may approach that of QM cluster models.^35,46−50^ QM cluster models may then be preferred, as they are often simpler to set up than QM/MM calculations.^35,51^ Different computational models, including small QM and large QM/MM models, have been shown to lead to divergent mechanistic pathways in catalysis.^52^ Making informed decisions about the appropriate computational approach to study the chemical and electronic properties of enzyme active sites is challenging. There is a lack of benchmarking studies in the literature addressing the optimal size of the QM region in QM/MM or comparing large QM models with QM/MM.^32,35,49,52−57^

**Figure 1:**
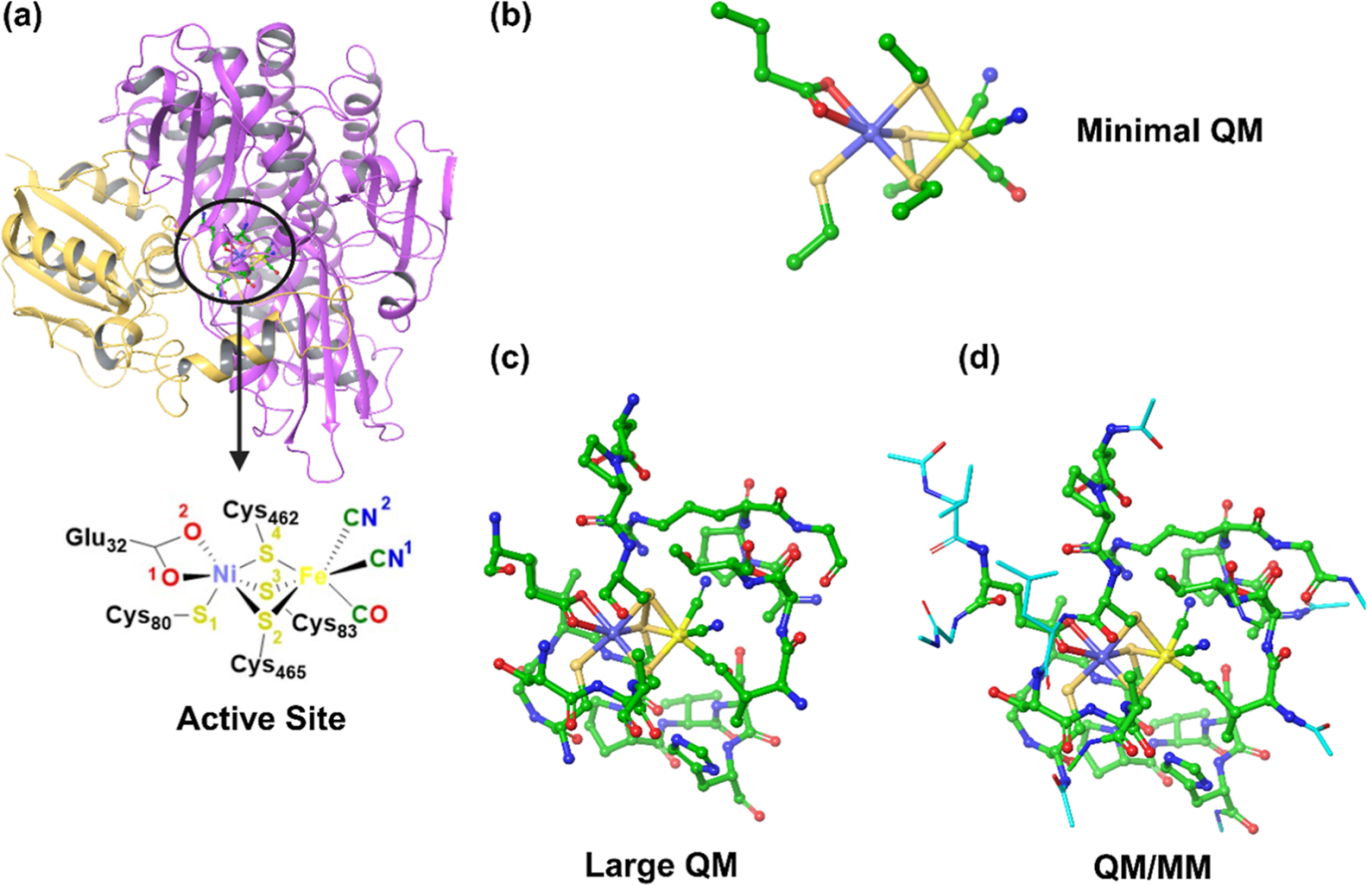
(a) Crystal structure of the fully oxidized state of [NiFe] hydrogenase from *H. thermoluteolus* SH (PDB: 5XF9) with an enlarged view of the active site. The two subunits are shown in yellow and pink. (b) Minimal enzyme active site QM used previously. (c,d) Large QM and QM/MM enzyme models used in the present study. The differences between these models are represented with distinct style for clarity.

In this work, we revisit the structural and electronic properties of the active site of the fully oxidized form of [NiFe] hydrogenase *Ht* SH by using computational methods. We employed a large QM cluster model (302 atoms) and a QM/MM model (392 atoms) to study the spin state energetic splitting among the closed-shell singlet (CS, S = 0), high-spin triplet, (HS, S = 1), and open-shell singlet broken symmetry (BS, S = 0) states (Figure 1). We critically investigate whether structural parameters of the active site are sensitive enough to the oxidation states of the heterobimetallic site to allow for the assignment of oxidation states at both the QM and QM/MM levels. The effect of the choice of model on the spin state splitting is also discussed for different exchange–correlation functionals. Analysis of the bonding interactions using both Natural Bond Orbital (NBO)^58,59^ and Intrinsic Bond Order (IBO)^60^ reveals that an unusual electron-deficient 3-center-2-electron bond is stabilizing the broken-symmetry over the high-spin state. This provides valuable insights into the electronic structure and orbital interactions. Our present work contributes to a deeper understanding of the stabilization of the superoxidized state of the enzyme. It also provides insights into the performance of different computational methods and levels of theory for studying metalloproteins.

## Computational Details

A large QM structural model of the enzyme was generated based on the crystal structure (5XF9).^30^ The coordinates of the active site and all amino acid residues with at least one atom located within 10 Å radius from nickel (E32, ^80^CGICPVSH^87^, ^394^APRG^397^, ^418^VST^420^, ^460^DPC^462^, and ^464^SCA^466^) were extracted (see Figure 1c). The system was protonated at pH 7 and the –CO and –NH groups with a dangling bond were saturated with hydrogen atoms, using the software Maestro 12.3.013. Standard protonation states were assigned to the side chains of E32, H87 (protonated at Nε), and D460. All cysteine residues that coordinate to the metals were deprotonated. This structure, hereafter referred to as the “QM model”, contains 302 atoms.

**Figure 2:**
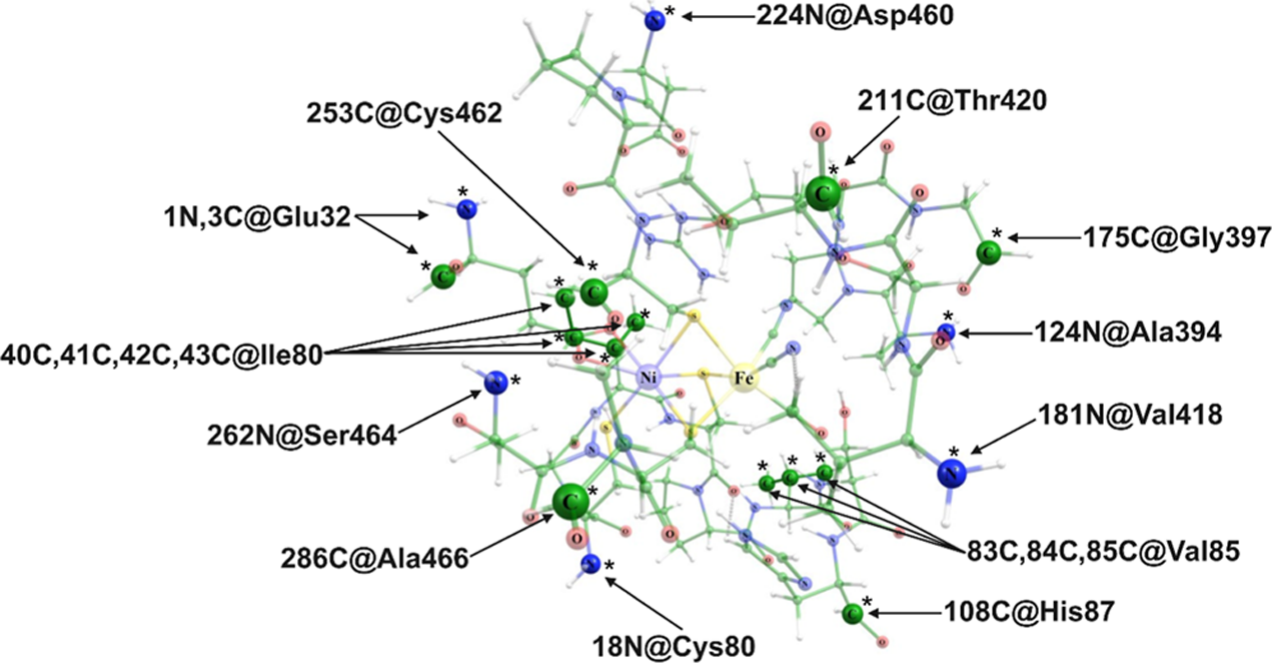
Large QM cluster of the fully oxidized state of *Ht* SH. Atoms constrained during structural optimizations are labeled and indicated by an asterisk.

The QM model was optimized at the DFT level using Turbomole 7.5.1.^56,61^ We optimized the CS (S = 0), the triplet (HS, S = 1), and the BS open-shell singlet (BS, S = 0) states. BS was obtained from HS by the spin-flipping Noodleman approach.^62^ The geometry optimizations were carried out under minimal constraints of selected remote atoms, ensuring that the overall active site structure is retained. The coordinates of 19 out of 302 atoms were fixed during optimizations: 1N,3C@Glu32; 18N@Cys80; 40C,41C,42C,43C@Ile82; 83C,84C,85C@Val85; 108C@His87; 124N@Ala394; 175C@Gly397; 181N@Val418; 211C@Thr420; 224N@Asp460; 253C@Cys462; 262N@Ser464; 286C@Ala466. These atoms correspond to the carboxylate carbons and amide nitrogen atoms at the position of truncated peptide bonds and to the carbon atoms of the side chains of Ile82 and Val85. The latter were fixed to avoid potential unrealistic interactions due to large movement of the side chains during structural optimization (see Figure 2).

The optimizations were performed with the BP86^63,64^ and PBE0^65,66^ functionals. The BP86 GGA functional tends to overstabilize the low-spin state due to underestimation of the electron correlation energy, while PBE0 is a hybrid functional that favors the high-spin state due to the incorporation of a 25% Hartree–Fock exchange.^67^ Thus, by using these two functionals, we made sure that our conclusions are not biased. Furthermore, these functionals have shown to offer a good balance between accuracy and computational cost for modeling metalloenzymes.^68−71^ The def2-SVP and def2-TZVP basis sets were used^72,73^ plus D3 dispersion corrections with Becke-Johnson damping (D3(BJ)) were applied.^74,75^ Initially, we optimized the structures with the smaller basis set with both functionals (BP86 and PBE0). To assess the effect of choice of basis set, we reoptimized the structures with PBE0/def2-TZVP. Since only a minute effect was observed (as discussed in detail later), the effect of the larger basis set with the BP86 functional on energetics was only evaluated in single point calculations. Relativistic effects were considered for the QM model by using the zero-order regular approximation.^76^ They did not affect structural parameters or energetic spin state splitting.

The optimized structures were compared to the crystal structure by calculating the heavy atom root-mean-square deviation (RMSD) and selected structural parameters. RMSD was used to assess the overall structural deviation from the crystal structure, while bonding parameters, including bond lengths and angles, were compared to show differences between the different metal oxidation states and electronic configurations of the active site. These comparisons helped in evaluating the accuracy of the model and the influence of oxidation states on the coordination environment of the active site. In addition, bonding orbital analysis was performed using NBO^69,77^ from ORCA 5.0.3.^78,79^ NBO analysis enabled the investigation of the electronic structure and bonding interactions in the active site. The NBO analysis provided insights into orbital interactions and delocalization effects, thus helping us to understand the bonding characteristics of the system.

The broken-symmetry (BS, S = 0) solution was obtained from the ferromagnetic HS (S = 1) upon spin flipping, and the magnetic exchange coupling constant *J* between the states was calculated using the Yamaguchi projection formula (eq 1).^80^ The *J*-value was calculated by using optimized structures.
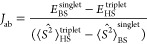
1

The spin-projected total energies of the BS states (*E*_BS_^APBS^) and the *J*-coupling constant were calculated for the spin-contaminated BP86 energies by the eqs 2–4 given by Yamaguchi^81^
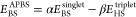
2
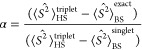
3
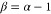
4

To further investigate the impact of Hartree–Fock exchange (HFX) on the magnetic coupling parameters, *J* coupling constants were calculated at various levels of HFX with the PBE0 functional and the def2-TZVP basis set. Structures optimized at the PBE0/def2-SVP level were used in single-point energy calculations with the ORCA 5.0.3 program.^78,79^ This analysis provided insights into how the electronic coupling is affected by the amount of exact exchange, putting the characterization of bonding and magnetic interactions of the system on solid ground.

**Figure 3:**
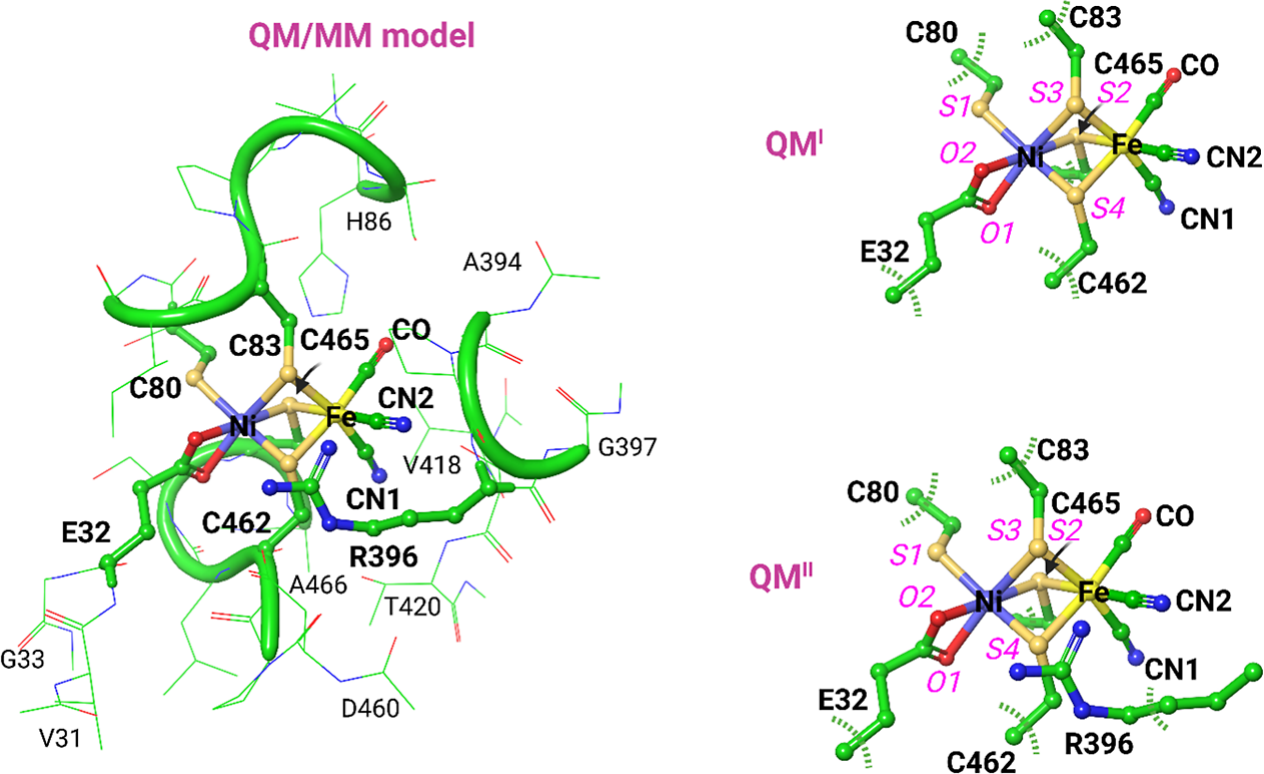
Enzyme structural model used in QM/MM calculations with the QM regions QM^I^ and QM^II^. QM/MM boundaries crossing through covalent bonds are shown by green dashed lines. Here, the orientation of the active site is reversed in comparison to other structural representations to clearly show R396 location, which is part of the QM^II^ region.

For comparison, QM/MM calculations were also performed, using an enzyme structural model which is slightly larger than the QM model.^82,83^ We hereafter refer to it as the “QM/MM model” (Figure 3), which besides the bioinorganic motif, comprises the following amino acid residues: ^30^RVEGH^34^, ^79^LCGICPVSHH^88^, ^393^EAPRGT^398^, ^417^IVSTT^421^, and ^459^FDPCLSCAT^467^. The N- and C-terminal residues of these sequences were truncated at the α carbon, and hydrogen atoms were added to complete their valences. Thus, our QM/MM model has a total of 392 atoms. As described below in more detail, the system was split into three regions: QM region, active region, and frozen region.

The QM/MM model was transferred to the ChemShell program (Tcl version 3.7)^84−86^ and optimized at the QM(DFT)/MM(CHARMM36) level using the def2-SVP and def2-TZVP basis sets.^87,88^ We individually optimized the CS, HS, and BS states. The effect of dispersion corrections was evaluated in single point calculations and optimizations with the def2-TZVP basis set,^72,73^ using Grimme’s dispersion correction.^74^ TURBOMOLE (version 7.2)^78^ and DL_POLY (version 4.09)^89^ were used as QM and MM codes, respectively. The calculations were performed with two different QM regions (QM^I^ and QM^II^). QM^I^ (33 atoms) consisted of atoms Ni, Fe, CO, and CN of the active site, the side chain of coordinating E32, and the side chains of cysteine residues coordinating to the transition metal ions. QM^II^ (45 atoms) extends beyond QM^I^ and also includes part of the side chain of R396 (Figure 3). This conserved arginine residue has been found to play a key role in [NiFe] hydrogenase catalysis,^90,91^ so we decided to evaluate here if inclusion of this residue in the QM region would lead to different results. The rest of the system was in the MM region. All optimizations were performed using the DL-FIND optimizer module of ChemShell and hybrid delocalized internal coordinates (HDLC).^92,93^ An electrostatic embedding scheme with charge shift correction was used to compute the electrostatic interaction between the QM region and the surrounding partial charges of the MM region.^94,95^ Valencies at the covalent bonds crossing the QM/MM boundary (i.e., Cα–Cβ bonds of cysteine residues and E32; Cγ–Cδ bond of R396) were saturated using hydrogen link atoms.^96^ All atoms were free to move during optimization, except the carbon atoms of the methyl groups of the terminii (acetyl and *N*-methyl amide) of amino acid residues. Thus, the MM region was split into active and frozen regions. When the QM^I^ region was used, the MM active and frozen regions have a total number of 349 and 10 atoms, respectively, and 337 and 10 atoms when using the QM^II^ region.

## Results and Discussion

### Active Site Structural Parameters Are Not Sensitive to Metal Oxidation and Spin States

**Figure 4:**
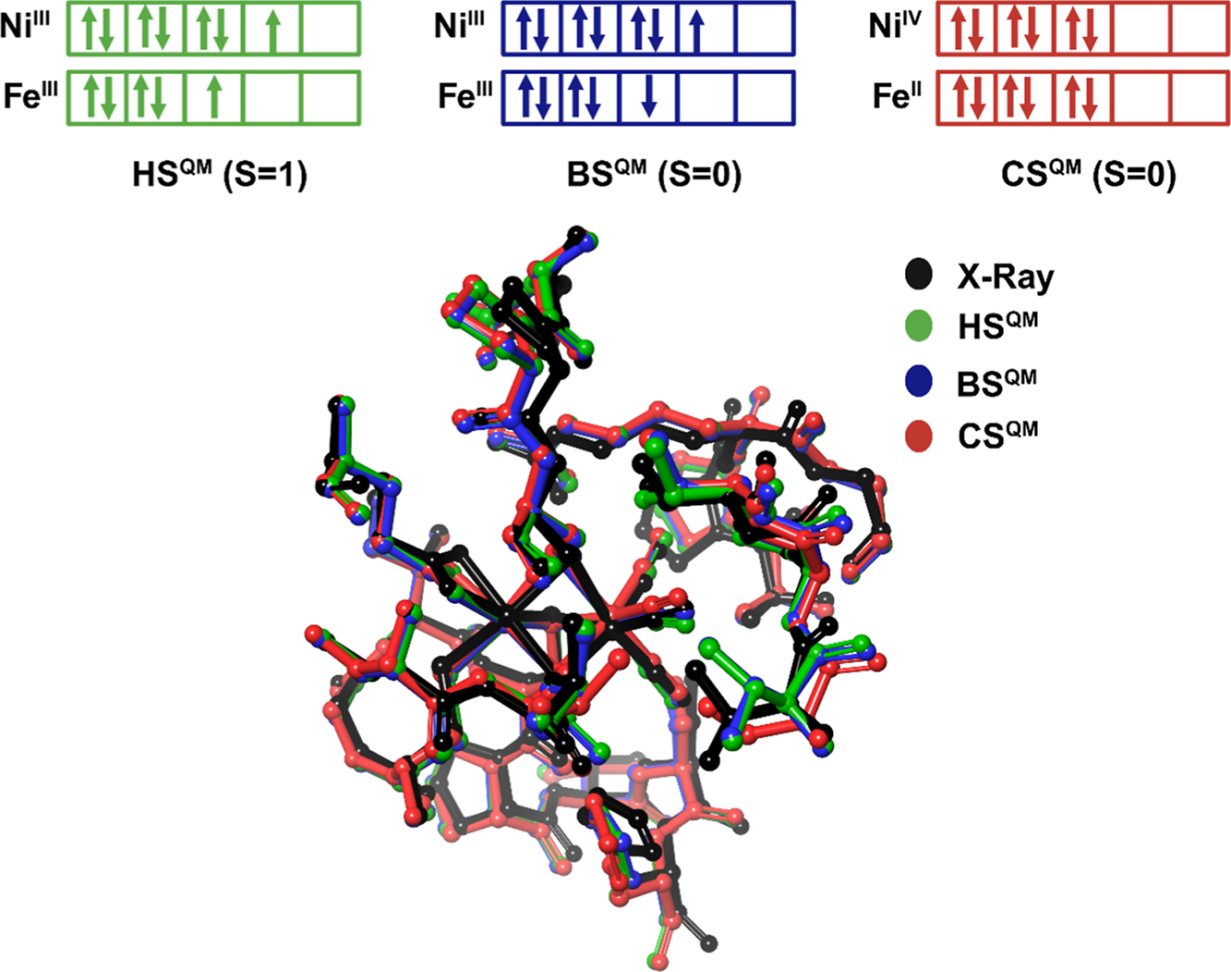
Superposition of large QM models of the active site of fully oxidized *Ht* SH in different spin states. Structurally optimized **HS^QM^**, **BS^QM^**, and **CS^QM^** models are compared to the X-ray structure.

Substrate binding, intermediate, and product states of [NiFe] hydrogenases are not easily accessible experimentally. For example, only at very high structural resolution of 0.89 Å, the bridging hydride has been resolved^97^ after its suggested binding mode by ENDOR^98^ and computations.^99^ Frequently, the Ni–Fe distance serves as an indicator of the oxidation states of the active site metals in X-ray structures of the Ni–A (2.80 Å), Ni–B (2.69 Å), and Ni–C (2.55 Å) states.^100,101^ We therefore analyzed the coordination modes of Ni and Fe in different oxidation and spin states of the active site and compared them with the available crystal structure (Figure 4). For the large QM model (Figure 2c), all calculations give a similar value for the Ni–Fe distance for all spin states. The maximum Ni–Fe distance difference observed between different spin states is of 0.08 Å, corresponding to the PBE0/def2-TZVP level of theory. The Ni–Fe distance values observed for **CS**^**QM**^ and **BS**^**QM**^ states are between 2.93 and 2.99 Å, which is 0.04–0.08 Å larger than those observed for **HS**^**QM**^ (2.88–2.91 Å). Thus, in terms of the Ni–Fe bond distance, **HS**^**QM**^ is closer to the crystal structure active site configuration, which shows a Ni–Fe distance of 2.86 Å at 2.58 Å resolution, and of considered to be of moderate to high quality (see Table 1). Dispersion corrections only have a very minor effect on the calculated Ni–Fe bond distance. Structures optimized with or without dispersion corrections (see PBE0/def2-SVP vs PBE0-D3(BJ)/def2-SVP in Table 1) are hardly distinguishable. Even scalar relativistic effects during the optimization (at zeroth order regular approximation level, ZORA^76^) do not significantly affect the calculated Ni–Fe distance: values of 2.88 Å for **HS**^**QM**^ and 2.93 Å for both **BS**^**QM**^ and **CS**^**QM**^ are obtained.

**Table 1:** Effect of Different Spin States, Basis Sets, and Dispersion Corrections on Selected Bond Distances (in Å) for Large QM Cluster and QM/MM Models of the Active Site, Compared to X-ray Structure of *Ht* SH

method	spin state	Ni–Fe	Ni–S2	Ni–S3	Ni–O1	Ni–O2
crystal structure		2.86	2.35	2.35	2.19	2.24
BP86/def2-SVP	CS	2.97	2.25	2.34	2.08	2.01
	HS	2.89	2.40	2.34	2.19	2.04
	BS	2.97	2.26	2.37	2.12	2.02
PBE0/def2-SVP	CS	2.94	2.22	2.28	2.02	1.97
	HS	2.89	2.27	2.59	2.14	1.97
	BS	2.93	2.27	2.56	2.15	1.97
PBE0-D3(BJ)/def2-SVP	CS	2.93	2.21	2.27	2.03	1.98
	HS	2.88	2.26	2.58	2.15	1.98
	BS	2.93	2.26	2.54	2.17	1.97
PBE0/def2-TZVP	CS	2.99	2.22	2.25	2.00	1.97
	HS	2.91	2.27	2.57	2.12	1.99
	BS	2.96	2.27	2.53	2.13	1.97
QMI(BP86, def2-SVP)/CHARMM36	CS	3.01	2.22	2.38	2.14	1.99
	HS	2.98	2.22	2.72	2.17	2.00
	BS	3.02	2.23	2.46	2.17	1.99
QMI(BP86, def2-TZVP)/CHARMM36	CS	3.03	2.21	2.37	2.16	2.00
	HS	3.05	2.20	2.83	2.19	2.00
	BS	3.04	2.21	2.42	2.19	2.00
QMI(PBE0, def2-SVP)/CHARMM36	CS	2.97	2.23	2.28	2.02	1.95
	HS	3.00	2.26	2.62	2.16	1.97
	BS	3.02	2.25	2.60	2.18	1.96
QMI(PBE0, def2-TZVP)/CHARMM36	CS	3.01	2.19	2.28	2.02	1.97
	HS	3.01	2.23	2.63	2.18	1.96
	BS	3.05	2.23	2.60	2.19	1.96
QMI(PBE0-D3, def2-TZVP)/CHARMM36	CS	3.01	2.18	2.27	2.03	1.97
	HS	2.98	2.23	2.60	2.19	1.96
	BS	3.02	2.23	2.57	2.21	1.95
QMII(PBE0, def2-SVP)/CHARMM36	CS	2.96	2.23	2.27	2.01	1.95
	HS	2.98	2.26	2.60	2.16	1.97
	BS	3.01	2.26	2.58	2.17	1.96
QMII(PBE0, def2-TZVP)/CHARMM36	CS	3.00	2.19	2.27	2.02	1.97
	HS	2.99	2.23	2.61	2.18	1.96
	BS	3.03	2.23	2.58	2.20	1.96

The bond distances of cysteine residues and inorganic small ligands (CO and CN) coordinating to Fe are very similar in all optimized spin states and differ by less than 0.06 Å. In contrast, some of the bond distances regarding Ni coordination show differences larger than 0.1 Å for different spin states (Tables 1 and S1 of the Supporting Information). For instance, the glutamate **Ni–O1** distances predicted for **HS**^**QM**^ are 0.11–0.12 and 0.01–0.07 Å larger compared to those obtained for **CS**^**QM**^ and **BS**^**QM**^, respectively, while the **Ni–O2** distance is the same or similar for all spin states (see Table 1).

The observed bidentate coordination of E32 to Ni in the crystal structure, where the bond distances between the carboxylate oxygens of E32 and Ni are similar (**Ni–O1** = 2.19 Å and **Ni–O2** = 2.24 Å), is only reproduced by **CS**^**QM**^. The BP86/def2-SVP **CS**^**QM**^ structure shows **Ni–O1** and **Ni–O2** distances of 2.08 and 2.01 Å, respectively. With the PBE0 functional, the difference between the two distances is even lower (≤0.05 Å), independent of the basis set and dispersion correction (Table 1). In contrast, **HS**^**QM**^ and **BS**^**QM**^ show **Ni–O1** bond distances, which are 0.10–0.20 Å larger than the calculated **Ni–O2** distances.

On the other hand, the calculated **Ni–O1** and **Ni–O2** distances obtained for **CS**^**QM**^ are 0.11–0.23 Å shorter than their values in the crystal structure (see Table 1). For **HS**^**QM**^ and **BS**^**QM**^, the **Ni–O1** distances are in good agreement with the crystal structure (2.12–2.19 Å vs 2.19 Å), whereas the **Ni–O2** distances are slightly reduced (1.97–2.04 Å vs 2.24 Å). Considering that the distances of an usual bidentate **Ni–O** coordination, as reported for different compounds involving the coordination of Ni to a carboxylate group, range between 1.97 and 2.27 Å,^102−104^ all DFT calculations give a bidentate coordination of E32 to Ni for all spin states. In the crystal structure, the temperature factor for residue E32 is 42.4 Å^2^ at 100 K and the carboxylate oxygen atoms have *B*-factors of 44.8 Å^2^ (OE1) and 41.7 Å^2^ (OE2), respectively. Although no alternative occupancies are explicitly given, this is indicative of a flexible or not unique coordination mode of E32.

Another important observation concerns the coordination of Ni to residues Cys465 (**S2**) and Cys83 (**S3**). The BP86 functional predicts the **Ni–S2** distance of **HS**^**QM**^ to be 0.15 and 0.14 Å larger than that of **CS**^**QM**^ and **BS**^**QM**^, respectively, and the **Ni–S3** distances to be similar for all optimized states (see Table 1). Furthermore, the **Ni–S2** distance of **HS**^**QM**^ is in closer agreement with the crystal structure (2.35 vs 2.40 Å). With PBE0, the situation is different. While the **Ni–S3** distances of **HS**^**QM**^ and **BS**^**QM**^ are 0.27–0.32 Å larger compared to those of **CS**^**QM**^, the **Ni–S2** distances are similar for all spin states. Furthermore, the **Ni–S3** distance of **CS**^**QM**^ is closer to the crystal structure value (see Table 1).

Thus, from our large QM cluster model calculations, only minor differences regarding coordination bond distances are observed among the optimized spin states. However, these do not allow for a definite assignment of metal oxidation states in the crystal structure. The small impact of dispersion correction on the structural parameters (Table 1) suggests that the dispersion effect is quite weak compared to the electronic effect. Analysis of bond angles, as reported in Table S1 of the Supporting Information, leads to the same conclusion.

Overall, the QM/MM optimized structures are very similar to the QM cluster model and display the same structural features concerning metallic center coordination (see Tables 1 and S1 of the Supporting Information). With the PBE0 functional, the difference between the Ni and coordinating E32 oxygen atoms **O1** and **O2** distances is <0.08 Å in **CS^QM/MM^**, 0.19–0.26 Å in **BS^QM/MM^**, and **HS^QM/MM^** (with **Ni–O1** > **Ni–O2**). Moreover, the **Ni–S3** distance in **CS**^**QM/MM**^ is 0.30–0.35 Å shorter than those in **BS**^**QM/MM**^ and **HS**^**QM/MM**^. No significant structural differences were detected when using the larger def2-TZVP basis set, dispersion correction, and the choice of QM^I^ vs QM^II^ regions. Again, this shows that dispersion has a minor role on structural parameters, while the electronic effect is dominating. This also reveals that the influence of enzyme residues beyond the first coordination sphere on the active site geometry is well described by the MM force field and is predominantly steric and electrostatic in nature. When using the BP86 functional in QM/MM calculation, distances **Ni–O1** > **Ni–O2** for all spin states. This is the most noticeable difference between the large QM and the QM/MM models. Nevertheless, also with the BP86 functional, the calculated distances indicate a close to bidentate coordination of residue Glu32 to Ni for both models.

Based on these results, it is impossible to accurately determine the oxidation state of the active site metallic center by a detailed comparative analysis of structural parameters only, and further criteria must be considered to allow a definite assignment.

### Energetic Splitting of Different Spin States of the Active Site

Since structural parameters were not conclusive regarding the assignment of metal oxidation states and even the GGA functional BP86 with a small basis set gave results in good agreement with X-ray structural data, more sophisticated electronic criteria were selected. The ordering and energetic spacing of the different spin states of the active site were investigated.

### Spin State Energetics in the Large QM Cluster Model of [NiFe] Hydrogenase

**Figure 5:**
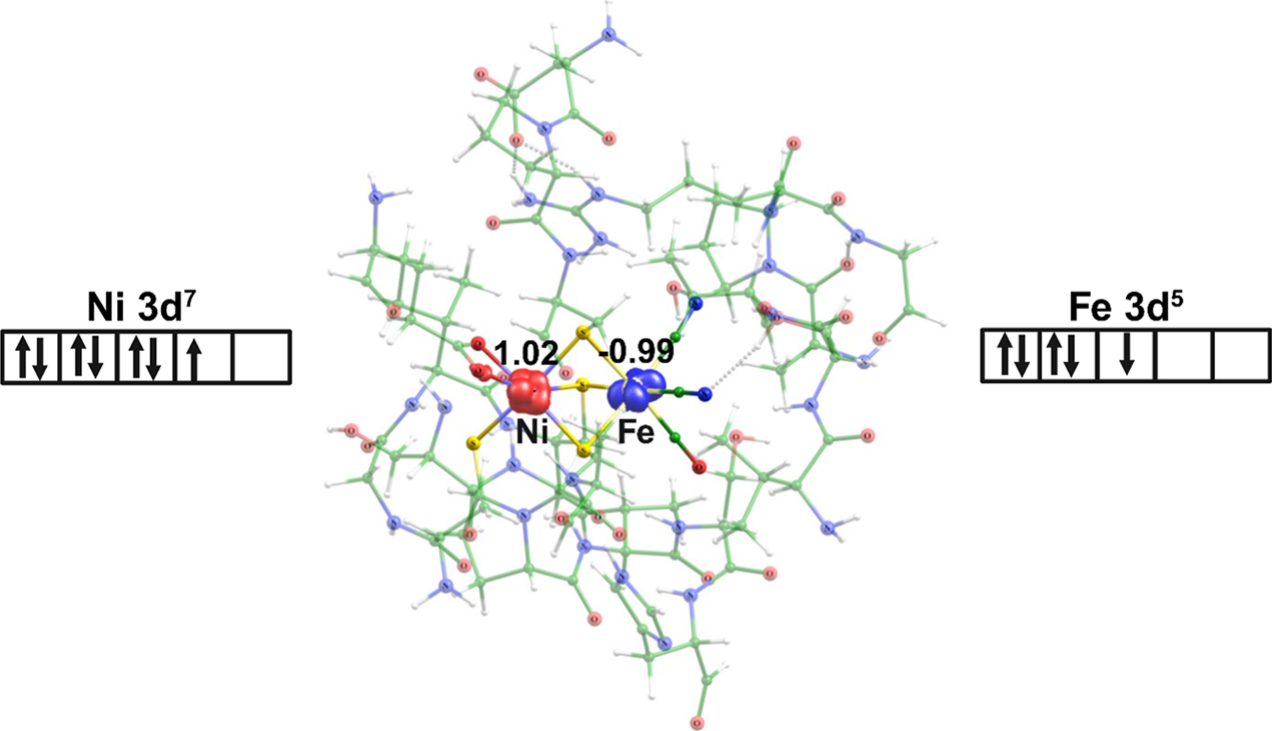
Spin density plot for the open-shell singlet BS^QM^ state (PBE0/def2-SVP) at a contour value of 0.03 au showing an antiferromagnetic coupling between low-spin Ni(III) (3d^7^, ↑) and low-spin Fe(III) (3d^5^, ↓).

The magnetic interaction between bridged binuclear transition metal complexes is characterized by the magnetic exchange coupling constant *J*. In an antiferromagnetic coupling of spins with opposite signs (*J* < 0), but also a ferromagnetic coupling (*J* > 0) may be feasible. Table 2 gives the calculated coupling constants and energy splitting for the scenarios discussed above. For the large QM model, the calculated *J*-values at BP86/def2-SVP and PBE0/def2-SVP optimized structures are −924 and −22 cm^–1^, respectively, with the negative sign indicating an antiferromagnetic coupling between low-spin Ni(III) (3d^7^, ↑) and low-spin Fe(III) (3d^5^, ↓). The spin density plot of such an antiferromagnetic coupling is shown in Figure 5. It can be clearly seen that the unpaired spin density is localized at both active site metals with the ↓-spin at the nickel and the ↓-spin electron at the iron atom (and vice versa for the energetically degenerate Ni^III^(↓)Fe^III^(↑) state).

**Table 2:** Spin State Splitting Energies of Large QM Models (in kJ/mol) of the BS and CS States Relative to the High Spin State (HS) and Calculated Exchange Coupling Constants *J* (cm^–1^)^c^

functional	basis set	closed shell	broken symmetry	*J*
BP86	def2-SVP opt	8.1	–17.1 (−11.2)^b^	–923.8 (−604.7)^a^
	def2-SVP D3(B)//def2-SVP^a^	25.2	–11.0 (−7.2)^b^	–595.3 (−390.0)^a^
	def2-TZVP D3(BJ)//def2-SVP^a^	11.1	–14.0 (−9.1)^b^	–754.6 (−494.0)^a^
PBE0	def2-SVP opt	61.5	–0.3	–22.4
	def2-TZVP D3(BJ)//def2-SVP^a^	49.6	–0.3	–23.5
	def2-SVP D3(BJ) opt	58.8	–0.8	–60.9
	def2-TZVP opt	72.8	0.4	35.4
	def2-TZVP D3(BJ)//def2-TZVP^a^	77.6	0.6	53.4

a Single point.

b After correction for spin contamination.

c Results after Corrections for Spin Contamination are given in Brackets.

With BP86, the **BS^QM^** state is the lowest in energy. At the BP86/def2-SVP level, **HS^QM^** is 17.1 kJ/mol (11.2 kJ/mol with AP-correction) higher in energy than **BS^QM^** and 8.1 kJ/mol lower in energy compared to **CS^QM^**. The energy gap between **CS^QM^** and **BS^QM^** is thus 25.2 kJ/mol (19.3 kJ/mol with AP-correction) (see Table 2) and increases to 36.2 kJ/mol (32.4 kJ/mol with AP correction) when dispersion corrections are applied. Dispersion corrections in combination with a larger basis set (def2-TZVP) give an energy gap of 25.1 kJ/mol (20.2 kJ/mol with the AP correction) (see Table 2).

The *J*-values were computed on optimized geometries of HS and BS. The large negative *J*-value of −924 cm^–1^ obtained with BP86 is overestimating due to spin contamination in BS. Its ⟨S^2^⟩ expectation value is 0.465, which is much lower than the theoretical 1.0, while for the PBE0/def2-TZVP BS optimized structure, it is 1.094.^105^ When *J* is calculated from differently optimized structures (HS and BS), the effect of dispersion corrections from the following single-point calculation is unrealistically large (see Table 2). The GGA BP86 functional relies solely on approximations of the exchange correlation energy, which can lead to inaccuracies in describing the exchange interactions, especially in open-shell systems.^106^ The hybrid PBE0 functional incorporates 25% of exact Hartree–Fock exchange into the exchange–correlation functional, which offers a better description of exchange interactions and reduces spin contamination.^107^ To correct the spin-contamination with the BP86 functional, we utilized the approximately spin-projected (AP) formula given by Yamaguchi (eqs 2–4).^81^ As a result, for BP86/def2-SVP, the *J*-value and the **HS-BS** energy gap value diminished to −604.7 cm^–1^ and −11.2 kJ/mol, respectively (see Table 2).

When the *J*-values were computed based on single-point energy calculations of the broken-symmetry state at the high-spin structure, spin contamination of the BS is significantly reduced (0.778). *J*-coupling is reduced to −288 cm^–1^ and dispersion corrections have no effect. As expected, dispersion corrections are relevant for structural parameters but do not contribute to the electronic interactions.

The hybrid PBE0 functional gives very consistent results, further supporting the consistency of our results. At PBE0/def2-SVP optimized structures, the **CS^QM^-BS^QM^** gap is 61.8 kJ/mol (58.8 kJ/mol with optimization including D3(BJ) dispersion correction), which is much higher than that obtained with BP86 (see Table 2). The gap increases to 72.4 kJ/mol for structures optimized with the larger def2-TZVP basis set. When the dispersion correction is applied using single-point calculations, the energy gap values are 49.9 and 77.0 kJ/mol, respectively (see Table 2). Thus, the PBE0 functional (with 25% HF exchange) also shows BS^QM^ to be lower in energy than CS^QM^. On the other hand, the energy difference between the **HS^QM^** and **BS^QM^** structures optimized at the PBE0/def2-SVP and PBE0/def2-TZVP levels are −0.3 kJ/mol (−0.8 kJ/mol with D3(BJ) optimization) and 0.4 kJ/mol, respectively, which suggest they are energetically degenerate states (see Table 2).

**Figure 6:**
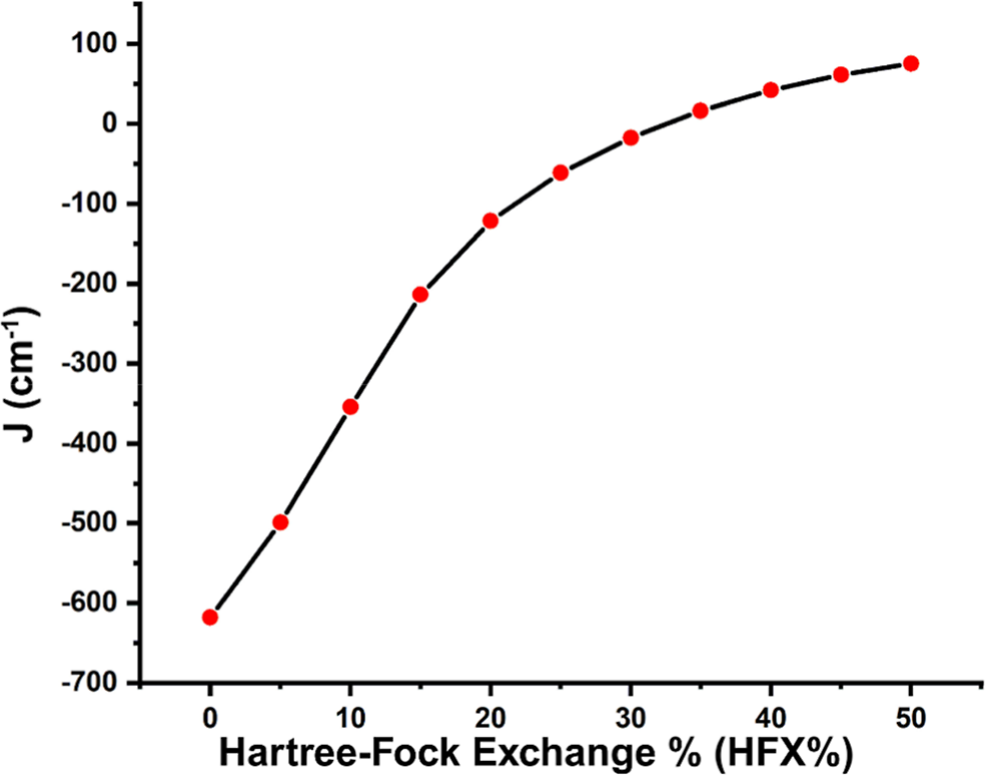
Effect of variation of Hartree–Fock exchange on the magnetic *J*-coupling (cm^–1^) (PBE0/def2-SVP).

When going from the GGA (BP86) to a hybrid (PBE0) functional, the stability of **BS^QM^** relative to **HS^QM^** decreases due to the inclusion of Hartree–Fock exchange. We have evaluated the effect of the variation of the amount of Hartree–Fock exchange (HFX) on the calculated *J*-values (see Figure 6). With no HFX, the *J*-value is found to be −618 cm^–1^ and decreases to −61 cm^–1^ with the standard 25% HFX in PBE0. This is similar to the results above for the two different functionals BP86 (no HFX, *J* = −604 cm^–1^) and PBE0 (25% HFX, *J* = −22 cm^–1^). As the Hartree–Fock exchange increases further, this stabilizes HS^QM^ even more and the *J*-value changes from negative to positive values, i.e., from antiferromagnetic to the ferromagnetic coupling (>35% HFX).

When the thermodynamic corrections from the minimal model are transferred to the large QM model of the present study, differences in Gibbs free energies (Δ*G*) for the **CS^QM^**-**BS^QM^** gap increase slightly to 37.9 kJ/mol from 36.2 kJ/mol at the BP86-D3(BJ)/def2-TZVP//BP86/def2-SVP level of theory and to 64.0 kJ/mol from 49.9 kJ/mol at the PBE0-D3(BJ)/def2-TZVP//PBE0/def2-SVP level of theory. This reinforces our conclusion on BS being the preferred state of the oxidized enzyme.

Whereas the choice of functional (BP86 and PBE0), size of basis sets (def2-SVP and def2-TZVP), and inclusion of dispersion corrections have some minor effect on structural parameters, spin state splitting and exchange coupling constants are less sensitive. However, the choice of the structural model for the BS solution is most critical, and the HS geometry is a good approximation for the BS state as well. In all of our results, the broken-symmetry state is lower in energy than the originally assigned closed-shell (S = 0) state.

### Spin State Energetics in QM/MM Representation of [NiFe] Hydrogenase

Our QM(PBE0)/(CHARMM36)MM calculations agree with the QM cluster model calculations using the PBE0 functional and suggest also that **BS^QM/MM^** and **HS^QM/MM^** are energetically degenerate. The QM/MM energy difference between these states ranges from 0.1 to 0.7 kJ/mol, depending on basis set, dispersion corrections, and use of QM region I vs II (see Table 3). **BS^QM/MM^** is slightly stabilized in comparison to **HS^QM/MM^** at all levels of theory. The calculated *J* coupling constants are between −62.8 and −11.7 cm^–1^ (again an antiferromagnetic coupling). When optimized using the QM^I^ region and the small def2-SVP basis set, the QM/MM calculated *J* (−22.1 cm^–1^) is almost identical to the QM cluster model. On the other hand, **CS^QM/MM^** is higher in energy than **BS^QM/MM^** and **HS^QM/MM^** independent of the level of theory, with energy differences between 69 and 97 kJ/mol, respectively (Table 3). Thus, the QM/MM calculations also support the assignment of a core [Ni^III^Fe^III^] oxidation state to be the ground state of oxidized [NiFe] hydrogenase from *Ht* SH. A CS state can be ruled out, according to our calculations.

**Table 3:** QM(DFT)/MM(CHARMM36) Energies (kJ/mol) of the BS and CS States Relative to the High Spin State (HS) and Calculated Exchange Coupling Constants *J* (cm^–1^)

QM region	functional	basis set	closed shell	broken symmetry	*J*^b^
I	BP86	def2-SVP	–5.9	–9.5	–540.7
		def2-TZVP	–12.3	–12.7	–649.3
	PBE0	def2-SVP	96.7	–0.2	–22.1
		def2-TZVP	70.9	–0.7	–52.4
		D3/def2-TZVP	69.6	–0.4	–33.5
		D3/def2-TZVP^a^	69.1	–0.6	–44.2
II	PBE0	def2-SVP	86.7	–0.1	–11.7
		def2-TZVP	70.4	–0.7	–62.8
		D3/def2-TZVP^a^	68.5	–0.3	–24.7

a Single point calculations.

b For separately optimized structures of HS and BS.

Concerning the BP86 functional, the same spin contamination issue observed with the QM model is present in the QM/MM model, leading to very high and unrealistic values of *J* (Table 3). The QM/MM model also indicates BS to be the most favored oxidation state and to be lower in energy than HS, by >9 kJ/mol. However, in contrast to the QM model, CS appears to be more stable than HS by >5 kcal/mol. Such a difference between the QM and QM/MM models and the spin contamination shows that the BP86 functional fails to provide a fully consistent picture of the spin states energetics and is outperformed by the hybrid PBE0 functional.

Both the QM and QM/MM results presented here are fully consistent and agree with those obtained by using a minimal QM model only (Figure 1). Our results thus indicate that either a minimal QM model or a QM/MM approach with a minimal QM region, in combination with a hybrid functional, such as PBE0, can provide a reliable description of redox and spin states of metals in [NiFe] hydrogenases active sites. Even with a small def2-SVP basis set, reliable structural parameters can be obtained plus a first qualitative insight into spin state splitting. Larger basis sets and the inclusion of dispersion corrections during optimizations can be used to further enhance the accuracy of calculated energetics. The applicability of only a minimal model also shows that the superoxidized state of the [NiFe] hydrogenase from *Ht* SH is an energetic minimum, and there are no positional constraints by the protein surrounding on the active site. Whereas the concept of an “entatic state” applies to the “rotated” structure of the active site in [FeFe] hydrogenases,^108,109^ this does not seem to apply for [NiFe] hydrogenases.^110,111^

### Stabilization of the Broken-Symmetry State from Orbital Analysis

The broken-symmetry (BS) electronic state was shown to be the lowest in energy in all DFT calculations using either QM or QM/MM models. Given the unusual coordination motif of the Ni atom by a terminal glutamate, one terminal cysteine residue, and three bridging cysteine residues, we performed an in-depth analysis of the chemical bonding of the large QM model. We applied NBO and IBO analyses, which provide the step from electron density to chemical concepts. They give an accurate description of the chemical bonding situation in molecules by breaking down the electron density into localized bonds and lone pairs. By quantifying these effects, a “chemical view” of molecular stability, reactivity, and electronic structure, especially in complex systems with delocalized or nontrivial bonding patterns, can be obtained.

In **CS^QM^**, a single density matrix is utilized for alpha (α) and beta (β) electrons, while in **BS^QM^**, separate density matrices are used for alpha (α) and beta (β) electrons. In NBO analysis, the density matrix of **CS^QM^** gives 12 standard 2-center 2-electron chemical bonds (2C–2e^–^), and a very similar binding situation of 2C–2e^–^ bonds are seen for the BS^QM^ α electron density matrix (see Table S2 of the Supporting Information). This is due to the same occupancy of α electrons in low-spin [Ni^IV^Fe^II^] **CS^QM^** [Ni(IV)-d^6^(3α,3β)-Fe(II)-d^6^(3α,3β)] and low-spin [Ni^III^Fe^III^] **BS^QM^** [Ni(III)-d^7^(3α,4β)-Fe(III)-d^5^(3α,2β)].

**Figure 7:**
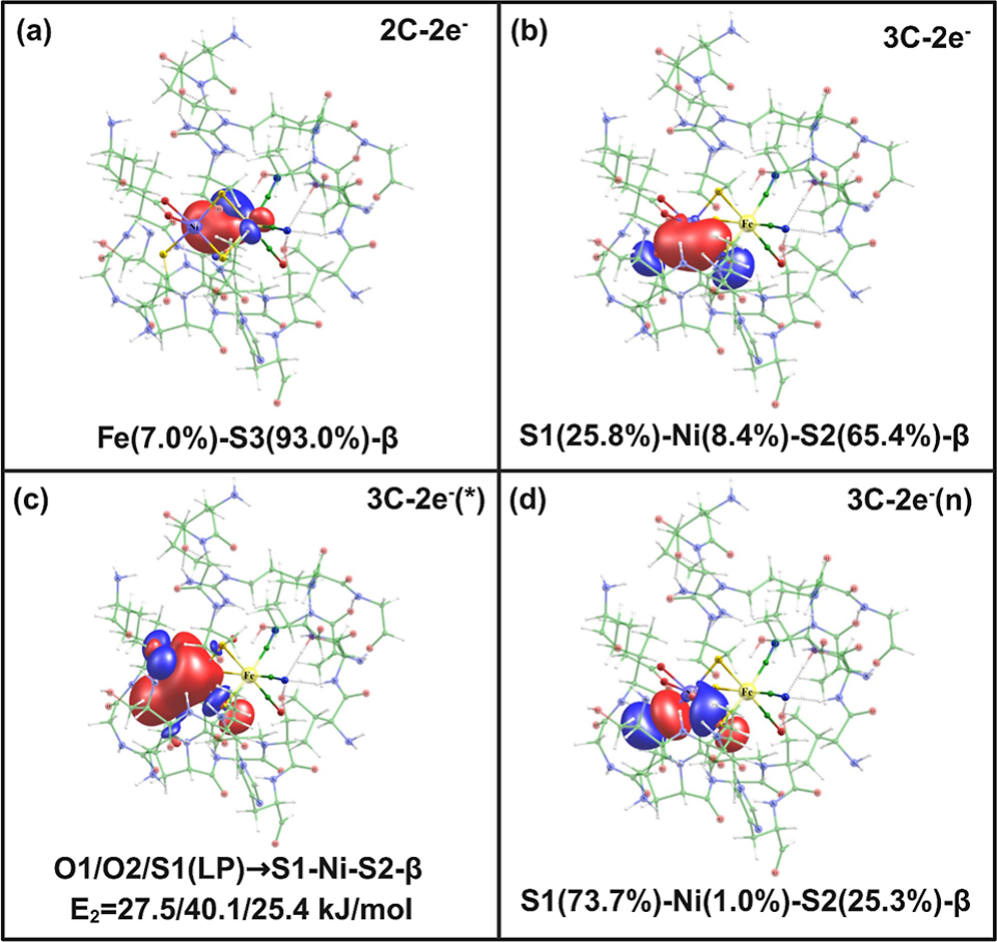
NBO orbitals showing unique bonding features in **BS^QM^** at a contour value of 0.03 au (a) 2-center 2-electron bonding between iron and Cys83 sulfur (Fe–S3, 2C–2e^–^), (b) 3-center 2-electron bonding between nickel and two Cys80 (S1) and Cys465 (S2) sulfur atoms (S1–Ni–S2, 3C-2e^–^), (c) three donor–acceptor bond from two Glu32 oxygen atoms (O1/O2) and one Cys80 sulfur (S1) to antibonding 3-center 2-electron S1–Ni–S2 orbital with respective second-order perturbation energy (*E*_2_) below (O1/O2/S1(LP) → S1–Ni–S2, 3C–2e^-–^(*)), and (d) 3-center 2-electron nonbonding orbital of S1–Ni–S2 (3C–2e^–^(n)).

The **BS^QM^**, however, exhibits distinct bonding orbitals because its β electron occupancy in the density matrix differs from that in the closed-shell state, leading to unique electronic interactions and bonding characteristics (see Figure 7). In **BS^QM^**, a dative ionic 2C–2e^–^ bond is formed between the iron and one bridging Cys83 sulfur (Fe–S3) with a 7.0% contribution from Fe and 93.0% from sulfur (see Figure 7a), which is not present in **CS^QM^** (αβ) (see Table S2). This reflects the change in the oxidation state from Fe(II) to Fe(III) and stabilization of the latter.

A relevant three-center two-electron (3C–2e^–^) bond is observed in **BS^QM^**, where two β electrons reside one each in bonding 3C and nonbonding 3C (3Cn). This delocalized 3C–2e^–^ bond is important for the stability and reactivity of certain chemical systems, particularly boron hydrides, carboranes, and other electron-deficient compounds.^112−115^ Understanding the role of the 3C–2e^–^ bond in biological systems is vital, as they influence the reactivity and stability of biomolecules.^116^ In biological systems, such a type of bond is observed in bridging hydride, agostic interactions, and dihydrogen complexes.^116−122^ It plays a crucial role in electron correlation, facilitating superexchange interactions between localized spins.^121^ The 3C–2e^–^ bond is also responsible for the stabilization of certain reaction intermediates and in mediating electron transfer processes.^119^ For instance, in cationic sulfur compounds, this bond facilitates electron communication, which is essential for various biochemical reactions.^123^ Despite its importance, direct spectral identification of molecules featuring the 3C–2e^–^ bond has been limited, highlighting the need for further research in this area.^123^

This bonding type involves three atomic centers sharing two electrons, which creates a highly stabilizing interaction in systems without enough electrons to form conventional 2C–2e^–^ covalent bonds. The 3C–2e^–^ bond between Cys80 sulfur (S1), Ni, and Cys465 sulfur (S2) is also a dative bond between nickel and sulfur atoms with a minor contribution (8.4%) from Ni and from S1 (25.8%) and S2 (65.8%) (see Figure 7b). The existence of this bond in BS was also identified and verified with the IBO approach (see Figure S2).^60^

In NBO analysis, second-order perturbation energy quantifies the stabilization due to electron delocalization from a donor (filled) orbital to an acceptor (empty) orbital, reflecting the magnitude of interactions such as hyperconjugation or charge transfer. The second-order perturbation energy (*E*_2_) indicates the donor–acceptor bond, also known as the dative bond (see Figure 7c). Here, the three terminal atoms (O1 and O2 from Glu32 and S1 from Cys80) close to the nickel center form a dative bond with the antibonding orbital of the 3C–2e^–^ bond, with energies of 27.5 kJ/mol (Glu32 oxygen O1), 40.1 kJ/mol (Glu32 oxygen O2), and 25.4 kJ/mol (Cys81 sulfur S1), respectively (see Figure 7c). The dative bond formed by Cys81 sulfur S1 is strong compared to the dative bonds formed by Glu32 oxygens O1 and O2. This is due to the more covalent character and larger orbital overlap tendency of sulfur lone-pair electrons compared with oxygen atoms. Both oxygen atoms, O1 and O2, of the coordinating Glu32 show similar magnitudes of the dative bond, indicating a bidentate coordination to the nickel center.

The nonbonding 3C–2e^–^ bond with the following contributions is present: Ni-1.0%, S1-73.7%, and S2-25.3% (see Figure 7d). Here, the electron is predominantly located at the S1 and S2 atoms due to the presence of higher electron density (Figure 7d). This nonbonding 3C–2e^–^ interaction contributes to the electron delocalization and provides stability to this **BS^QM^** state.

The presence of this delocalized three-center two-electron bond (bonding, antibonding, and nonbonding) (see Figure 7b–d) in **BS^QM^** determines the stability of this state compared to **CS^QM^**, where only standard 2C–2e^–^ bonds are present (see Table S2). The lowest energy and unusual 3C–2e^–^ bond of **BS^QM^** strongly supports the observed uncommon structural change of the enzyme active site in the oxidative environment. This avoids direct oxygen binding to the nickel atom of the active site or a cysteine residue oxidation.^100,101^ A similar binding situation can also be observed for biomimetic models of the hydrogenase active site with a bridging atom.^124^

## Conclusions

In this work, we studied the structural and electronic properties of the active site of oxidized [NiFe] hydrogenase from *H. thermoluteolus* SH using a large QM cluster and QM/MM calculations. We found that the structures of the CS (S = 0), triplet (HS, S = 1), and open-shell singlet BS (S = 0) spin states of the oxidized enzyme are very similar in terms of bond distances and angles. Therefore, they all resemble the experimental structural features, but this does not allow us to assign a definite spin state to the metals in the X-ray structure. However, the BS state is lower in energy than the CS state in all of our QM and QM/MM calculations, making it the electronic ground state of the fully oxidized enzyme. Detailed NBO analysis reveals an unusual 3C–2e^–^ bond in the BS state, which is not present in the CS state. The presence of 3C–2e^–^ bonds and the lower energy of the broken-symmetry state in the oxidative environment are key factors in its energetic stabilization, which protects the active site from forming inactive species in the presence of oxygen and cysteine oxidation. This contributes to the oxygen tolerance of *Ht* SH, which is a novel aspect of its function. The trigger of the large conformational change and structural dynamics when going from a Ni(III)Fe(II) to a Ni(III)Fe(III) state remains to be investigated. Our large QM and QM/MM models provide consistent results for this enzyme and its oxidation states, allowing us to establish realistic enzyme models. A high-valent Ni(IV) oxidation state is not the lowest energy state, and our calculations rule it out as a possibility. Since the conclusions from the minimal QM cluster model are still valid, polarization, steric effects, and conformational restrictions from residues beyond the first sphere of coordination are not significant factors in this case. The electronic structure and formal oxidation states of the heterobimetallic active site and its first coordination sphere residues are critical factors that need to be treated accurately to obtain reliable results. The coordination of the catalytically active nickel atom by a terminal glutamate in the fully oxidized Ni(III)Fe(III) state is an interesting aspect of the dynamics of coordination in [NiFe] hydrogenases. In standard [NiFe] hydrogenase enzymes, the iron atom in the active site is not redox active and remains in an EPR-silent Fe(II) low-spin state. The concept of a third μ-bridging cysteine ligand mediating the electronic exchange between the two metals and enabling the Fe(II) to Fe(III) oxidation is a novel concept for hydrogenase enzymes. This opens several avenues for further research on enzymatic oxygen tolerance and the design of biomimetic catalysts with enhanced stability.

## Supporting Information Available

Electronic energies of all species, detailed structural parameters such as bond lengths and angles of large QM and QM/MM models, NBO and IBO analysis of QM^CS^ and QM^BS^, and Cartesian coordinates of all species (PDF)
