# Supplementary material for: Fully Oxidized State of the Oxygen-Tolerant [NiFe] Hydrogenase from Hydrogenophilus thermoluteolus SH: A Quantum Mechanics Cluster and Quantum Mechanics/Molecular Mechanics Study
Source: Inorg Chem. 2025 May 7;64(19):9558–70. doi: 10.1021/acs.inorgchem.5c00503 (PMC12093298; doi:10.1021/acs.inorgchem.5c00503)
Supplement: Supplementary file 1 — ic5c00503_si_001.pdf [file ic5c00503_si_001.pdf]

Supporting Information

Fully Oxidized State of the Oxygen-Tolerant [NiFe] Hydrogenase from *Hydrogenophilus thermoluteolus* SH: A Quantum Mechanics Cluster and Quantum Mechanics/Molecular Mechanics Study

Ravi Kumar, Andrés M. Escorcia, Matthias Stein*

Max Planck Institute for Dynamics of Complex Technical Systems, Molecular Simulations and Design Group, Sandtorstrasse 1, 39106 Magdeburg, Germany.

*Email: matthias.stein@mpi-magdeburg.mpg.de

RMSD values:

We computed the heavy atom RMSD between the optimized structures and the X-ray structure. The QM cluster model structures of HS (HS^{QM}), BS (BS^{QM}) and CS (CS^{QM}) optimized with BP86/def2-SVP showed RMSD values of 0.631 Å, 0.629 Å and 0.625 Å, respectively. Similar values were obtained for structures optimized with PBE0/def2-SVP (0.675 Å, 0.676 Å and 0.681 Å). Thus, HS^{QM}, BS^{QM} and CS^{QM} show only a minor difference in RMSD value of < 0.006 Å with both functionals, which shows that the structural differences between these states are subtle. Based on the RMSD values, which offer a global structural comparison, the prevalent oxidation states of the metals in the crystal structure cannot be firmly assigned (see **Figure 4 in the manuscript**).

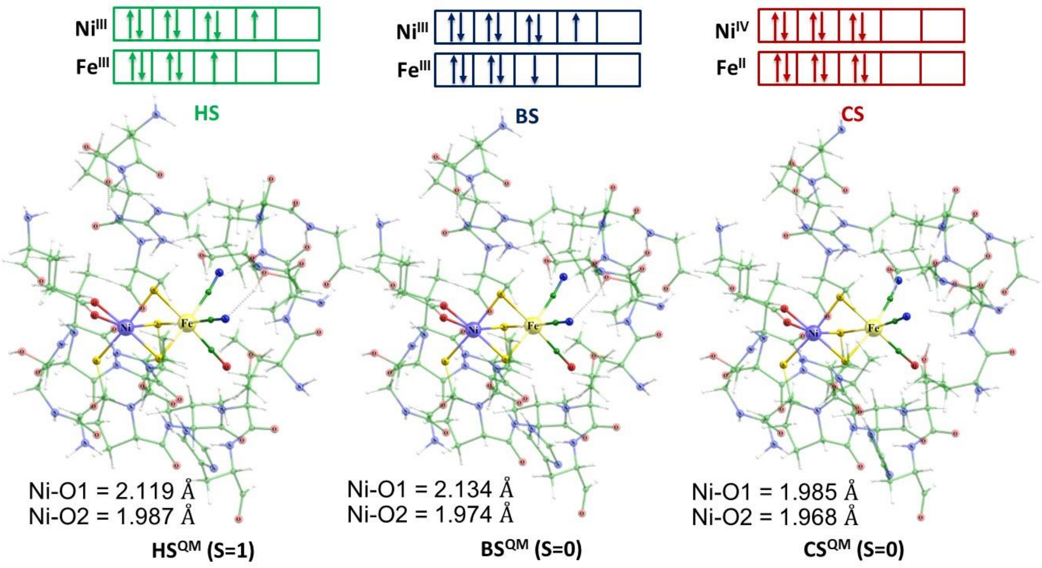

Figure S1. DFT (PBE0/def2-TZVP) optimized structures of triplet (HS^{QM}, S=1), broken symmetry singlet (BS^{QM}, S=0) and closed shell singlet (CS^{QM}) states of the oxidized state of the [NiFe] hydrogenase active site.

Table S1: Bond lengths and angles describing the coordination modes of Ni and Fe in the X-Ray structure, and the structures optimized in the present study at different levels of theory.

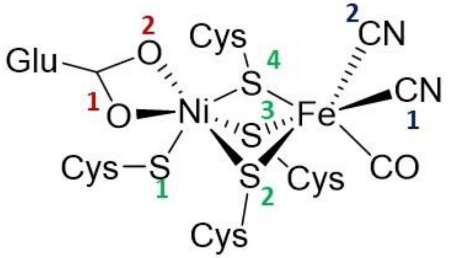

Method	Species	Ni-Fe	Ni-S1	Ni-S2	Ni-S3	Ni-S4	Ni-O1	Ni-O2	Fe-CN1	Fe-CN2	Fe-CO	Fe-S2	Fe-S3	Fe-S4
		**Bond lengths (Å)**												
**X-Ray[20]**		2.86	2.36	2.35	2.35	2.34	2.19	2.24	1.77	1.80	1.77	2.35	2.35	2.35
**BP86/def2-SVP**	**CS**	2.97	2.27	2.25	2.34	2.29	2.08	2.01	1.86	1.88	1.74	2.38	2.30	2.39
	**HS**	2.89	2.26	2.40	2.34	2.37	2.19	2.04	1.86	1.88	1.74	2.40	2.34	2.37
	**BS**	2.97	2.26	2.26	2.37	2.29	2.12	2.02	1.86	1.88	1.74	2.39	2.31	2.39
**PBE0/def2-SVP**	**CS**	2.94	2.26	2.22	2.28	2.28	2.02	1.97	1.86	1.88	1.76	2.35	2.29	2.35
	**HS**	2.89	2.25	2.27	2.59	2.27	2.14	1.97	1.89	1.91	1.81	2.37	2.26	2.33
	**BS**	2.93	2.25	2.27	2.56	2.27	2.15	1.97	1.90	1.90	1.81	2.38	2.25	2.34
**PBE0-D3(BJ)/def2-SVP**	**CS**	2.93	2.26	2.21	2.27	2.26	2.03	1.98	1.85	1.87	1.76	2.33	2.29	2.33
	**HS**	2.88	2.25	2.26	2.58	2.25	2.15	1.98	1.89	1.89	1.81	2.35	2.26	2.31
	**BS**	2.93	2.25	2.26	2.54	2.26	2.17	1.97	1.89	1.89	1.81	2.36	2.24	2.32
**PBE0-D3(BJ)/def2-SVP(ZORA)***	**CS**	2.93	2.25	2.21	2.26	2.26	2.01	1.97	1.84	1.87	1.75	2.33	2.28	2.33
	**HS**	2.88	2.24	2.25	2.59	2.25	2.13	1.97	1.88	1.89	1.80	2.34	2.25	2.31
	**BS**	2.93	2.24	2.25	2.55	2.25	2.15	1.95	1.88	1.88	1.79	2.35	2.23	2.32
**PBE0/def2-TZVP**	**CS**	2.99	2.26	2.22	2.25	2.26	2.00	1.97	1.89	1.90	1.77	2.35	2.30	2.34
	**HS**	2.91	2.24	2.27	2.57	2.26	2.12	1.99	1.92	1.91	1.82	2.36	2.27	2.32
	**BS**	2.96	2.24	2.27	2.53	2.26	2.13	1.97	1.92	1.91	1.82	2.37	2.25	2.33
**QMI(BP86, def2-SVP)/CHARMM36**	**CS**	3.01	2.26	2.22	2.38	2.28	2.14	1.99	1.88	1.88	1.74	2.35	2.26	2.42
	**HS**	2.98	2.24	2.22	2.72	2.25	2.17	2.00	1.89	1.90	1.76	2.36	2.29	2.38
	**BS**	3.02	2.25	2.23	2.46	2.27	2.17	1.99	1.88	1.89	1.75	2.37	2.26	2.42

**QMI(BP86, def2-TZVP)/CHARMM36**	**CS**	3.03	2.26	2.21	2.37	2.27	2.16	2.00	1.89	1.88	1.76	2.33	2.25	2.40
	**HS**	3.05	2.23	2.20	2.83	2.24	2.19	2.00	1.90	1.90	1.77	2.34	2.26	2.37
	**BS**	3.04	2.25	2.21	2.42	2.26	2.19	2.00	1.89	1.89	1.76	2.34	2.25	2.40
**QMI(PBE0, def2-SVP)/CHARMM36**	**CS**	2.97	2.26	2.23	2.28	2.28	2.02	1.95	1.89	1.89	1.76	2.33	2.30	2.40
	**HS**	3.00	2.24	2.26	2.62	2.26	2.16	1.97	1.90	1.91	1.81	2.35	2.29	2.34
	**BS**	3.02	2.24	2.25	2.60	2.26	2.18	1.96	1.90	1.91	1.80	2.36	2.28	2.35
**QMI(PBE0, def2-TZVP)/CHARMM36**	**CS**	3.01	2.26	2.19	2.28	2.25	2.02	1.97	1.89	1.90	1.77	2.30	2.29	2.36
	**HS**	3.01	2.24	2.23	2.63	2.24	2.18	1.96	1.90	1.92	1.81	2.33	2.27	2.32
	**BS**	3.05	2.24	2.23	2.60	2.25	2.19	1.96	1.90	1.92	1.81	2.34	2.26	2.34
**QMI(PBE0-D3, def2-TZVP)/CHARMM36**	**CS**	3.01	2.26	2.18	2.27	2.25	2.03	1.97	1.88	1.90	1.77	2.30	2.29	2.35
	**HS**	2.98	2.23	2.23	2.60	2.24	2.19	1.96	1.90	1.92	1.81	2.32	2.28	2.32
	**BS**	3.02	2.23	2.23	2.57	2.25	2.21	1.95	1.90	1.92	1.81	2.34	2.26	2.33
**QMII(PBE0, def2-SVP)/CHARMM36**	**CS**	2.96	2.27	2.23	2.27	2.29	2.01	1.95	1.88	1.88	1.76	2.33	2.30	2.41
	**HS**	2.98	2.25	2.26	2.60	2.27	2.16	1.97	1.90	1.90	1.80	2.34	2.29	2.35
	**BS**	3.01	2.25	2.26	2.58	2.27	2.17	1.96	1.90	1.90	1.80	2.35	2.28	2.36
**QMII(PBE0, def2-TZVP)/CHARMM36**	**CS**	3.00	2.26	2.19	2.27	2.26	2.02	1.97	1.89	1.89	1.77	2.29	2.29	2.37
	**HS**	2.99	2.24	2.23	2.61	2.25	2.18	1.96	1.90	1.91	1.81	2.32	2.27	2.33
	**BS**	3.03	2.24	2.23	2.58	2.26	2.20	1.96	1.91	1.91	1.81	2.33	2.26	2.35
**Bond Angles (°)**														

Method	Species	Ni-S2-Fe	Ni-S3-Fe	Ni-S4-Fe	O1-Ni-O2	CN1-Fe-CN2	S4-Fe-CO	S3-Fe-CN1	S2-Fe-CN2	S2-Ni-O2	S3-Ni-O1	S4-Ni-S1
**X-Ray[20]**		75.2	75.1	75.2	60.1	91.0	165.8	161.0	169.3	175.9	152.4	168.4
**BP86/def2-SVP**	**CS**	79.8	79.6	78.8	64.8	93.1	178.9	168.8	169.9	176.3	162.1	169.9
	**HS**	76.6	73.6	76.9	62.8	92.9	177.2	175.1	173.4	173.5	157.7	169.6
	**BS**	79.3	78.7	78.6	63.9	93.1	178.8	170.9	170.4	175.5	160.5	170.6
**PBE0/def2-SVP**	**CS**	79.9	80.1	78.7	65.4	95.3	178.1	167.8	169.2	177.1	161.7	171.2
	**HS**	77.1	72.6	77.8	63.7	89.1	177.1	174.2	175.9	175.2	158.1	168.9
	**BS**	78.2	74.8	78.9	63.6	89.9	176.7	173.1	174.7	175.7	159.0	169.6
**PBE0-D3(BJ)/def2-SVP**	**CS**	79.9	80.1	78.7	65.4	95.3	178.1	167.8	169.2	177.1	161.7	171.2
	**HS**	77.1	72.6	77.8	63.7	89.1	177.1	174.2	175.9	175.2	158.1	169.6
	**BS**	78.2	74.8	78.9	63.6	89.9	176.7	173.1	174.6	175.6	159.0	169.6
**PBE0-D3(BJ)/def2-SVP(ZORA)***	**CS**	80.5	80.2	79.4	65.7	94.4	177.7	166.5	169.5	177.7	161.4	171.6
	**HS**	77.7	72.7	78.4	64.0	87.8	176.8	172.7	175.6	175.8	157.3	169.2
	**BS**	79.0	75.2	79.7	63.9	88.8	176.2	171.8	174.4	176.3	158.2	170.1
**PBE0/def2-TZVP**	**CS**	81.7	82.1	80.8	65.9	92.5	177.8	168.3	168.4	178.3	16.3.1	171.1
	**HS**	77.9	73.7	78.9	63.7	87.6	176.2	176.1	175.8	176.4	158.1	169.4
	**BS**	79.2	76.1	80.2	63.6	88.2	176.3	175.5	174.4	176.9	158.9	169.9
**QMI(BP86, def2-SVP)/CHARMM36**	**CS**	82.2	80.8	79.5	64	90.8	177.3	164	176.7	175	161.5	171.5
	**HS**	81.2	72.5	80.1	63.4	86.4	174.2	169.9	173.8	174.6	159	166
	**BS**	82.1	79.5	80.2	63.6	90.2	176.3	164.9	176.9	174.8	161.6	170.3
**QMI(BP86, def2-TZVP)/CHARMM36**	**CS**	83.9	82.1	81	63.4	91	177.7	163.8	176.8	176.4	161.3	171.7
	**HS**	84.2	72.5	82.7	63.1	86.6	174.2	171.1	174.4	176.2	158.7	164.9
	**BS**	83.8	81.3	81.4	63	90.6	176.8	164.2	177.1	176.1	161.3	171
**QMI(PBE0, def2-SVP)/CHARMM36**	**CS**	81.3	80.8	78.8	65.8	93.4	178.2	165.0	174.5	176.5	161.2	174.3
	**HS**	81.3	74.8	81.3	63.3	87.4	173.3	169.7	175.4	174.6	160.4	169.9
	**BS**	81.9	76.2	81.9	63.3	87.9	173.9	168.9	176.1	174.8	160.7	170.2
**QMI(PBE0, def2-TZVP)/CHARMM36**	**CS**	84.3	82.5	81.6	65.2	92.2	176.4	163.1	176.4	176.3	163	172.8

	**HS**	82.8	75.3	82.5	63.1	87.4	173.3	169.9	175.3	175.9	160.2	169.8
	**BS**	83.7	77.2	83.2	63.0	88.1	173.8	168.6	176.3	176.1	160.8	170.3
**QMI(PBE0-D3, def2-TZVP)/CHARMM36**	**CS**	84.2	82.4	81.5	65.2	92	177	163.2	176.5	176.4	162.7	172.6
	**HS**	81.9	75.2	81.6	62.9	87	173.3	170.1	174.7	175.4	160	170.4
	**BS**	82.7	77	82.4	62.8	87.6	173.5	168.9	175.7	175.5	160.4	170.8
**QMII(PBE0, def2-SVP)/CHARMM36**	**CS**	81.1	80.8	78.1	66	93.7	177.9	165.1	173.6	176.9	160.6	174.6
	**HS**	80.7	74.7	80.2	63.3	87.8	174	169.6	176.1	173.8	160.9	170.2
	**BS**	81.4	76.1	80.9	63.3	88.4	174.5	168.8	176.8	174.0	161.3	170.5
**QMII(PBE0, def2-TZVP)/CHARMM36**	**CS**	84.2	82.4	80.8	65.3	92.6	176.4	163	175.5	175.8	163.33	172.7
	**HS**	82.2	75.3	81.5	63.0	87.7	173.8	169.8	176	175.2	160.8	170.2
	**BS**	83.2	77.4	82.4	62.9	88.6	174.6	168.4	177	175.4	161.4	170.6

Table S2: NBO analysis of the BS and CS states of the large QM model (PBE0/def2-SVP).

S.No.	Broken symmetry (open-shell) singlet state				Closed-shell singlet state	
	Orbital Diagram	α	Orbital Diagram	β	Orbital Diagram	αβ(paired)
1	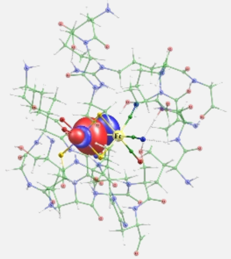	Ni(14.1%)-S3(85.9%)-α	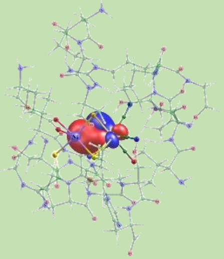	Fe(7.0%)-S3(93.0%)-β	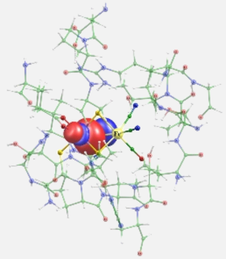	Ni(35.3%)-S3(64.7%)
2	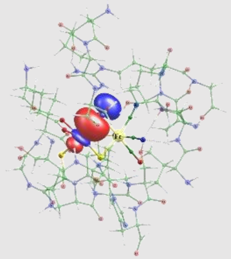	Ni(20.2%)-S4(79.8%)-α	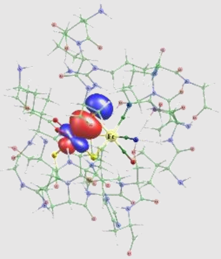	Ni(47.1%)-S4(52.9%)-β	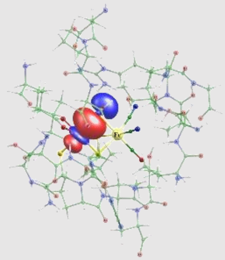	Ni(34.8%)-S4(65.2%)
3	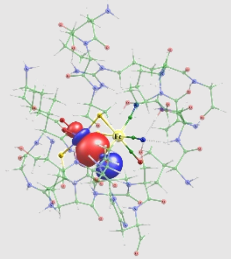	Ni(27.6%)-S2(82.7%)-α	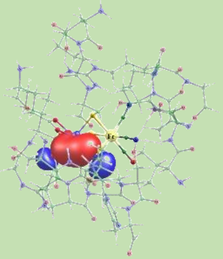	S1(25.8%)-Ni(8.4%)-S2(65.8%)-β (3C)	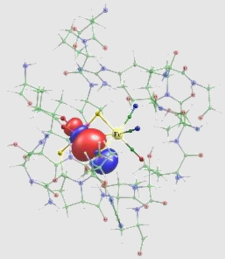	Ni(33.3%)-S2(66.7%)
4	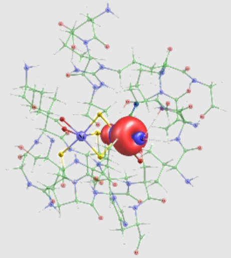	Fe(31.9%)-C1(68.1%)-α	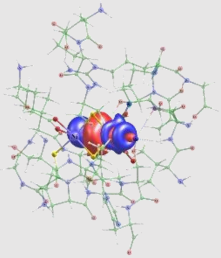	Fe(28.1%)-C1(71.9%)-β	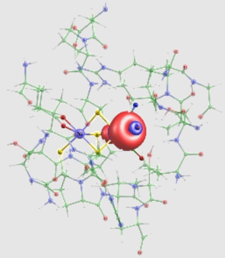	Fe(25.9%)-C1(74.1%)

5	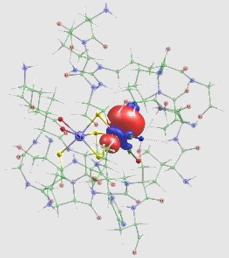	Fe(33.0%)-C2(66.9%)-α	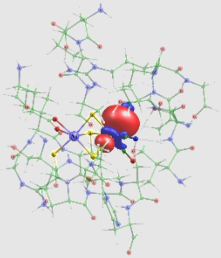	Fe(28.9%)-C2(71.1%)-β	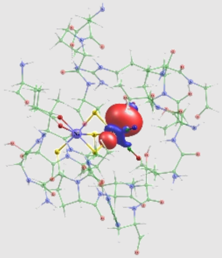	Fe(26.6%)-C2(73.4%)
6	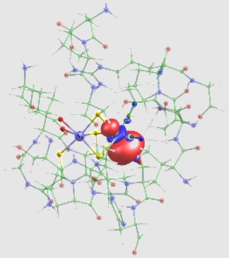	Fe(28.9%)-C(71.1%)-α	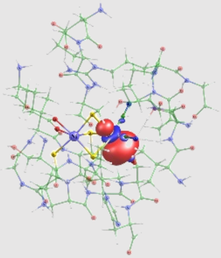	Fe(25.8%)-C(74.2%)-β	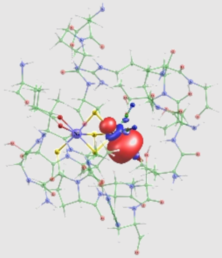	Fe(25.0%)-C(75.0%)
7	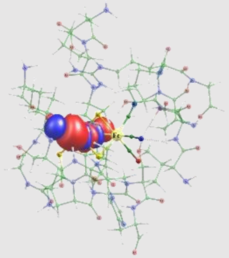	O1(LP)→Ni-S3(BD*)-α E_2=58.6 kJ/mol	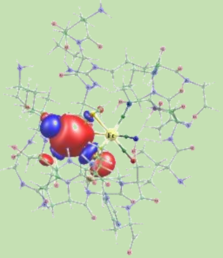	O1(LP)→S1-Ni-S2(3C*)-β E_2=27.5 kJ/mol	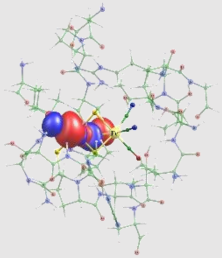	O1(LP)→Ni-S3(BD*) E_2=191.6 kJ/mol
8	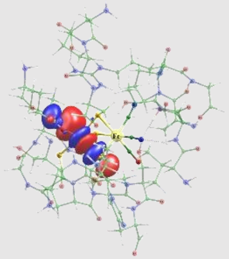	O2(LP)→Ni-S2(BD*)-α E_2=82.8 kJ/mol	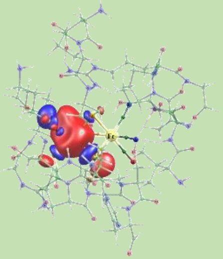	O2(LP)→S1-Ni-S2(3C*)-β E_2=40.1 kJ/mol	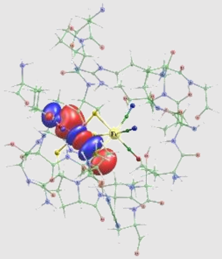	O2(LP)→Ni-S2(BD*) E_2=175.7 kJ/mol

9	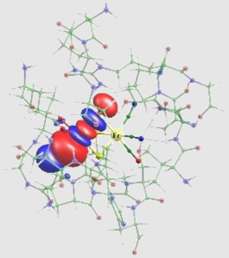	S1(LP)→Ni-S4(BD*)-α<br>E_2=176.5 kJ/mol	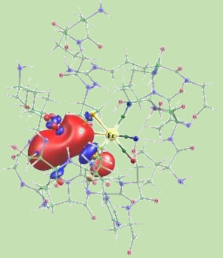	S1(LP)→S1-Ni-S2(3C*)-β<br>E_2=25.4 kJ/mol	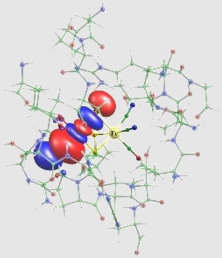	S1(LP)→Ni-S4(BD*)<br>E_2=419.7 kJ/mol
10	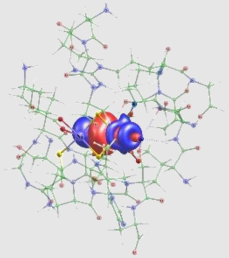	S3(LP)→Fe-C1(BD*)-α<br>E_2=205.8 kJ/mol	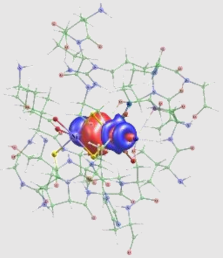	S3(LP)→Fe-C1(BD*)-β<br>E_2=193.7 kJ/mol	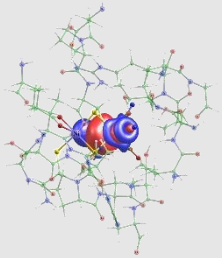	S3(LP)→Fe-C1(BD*)<br>E_2=323.8 kJ/mol
11	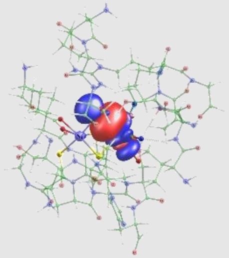	S4(LP)→Fe-C(BD*)-α<br>E_2=175.7 kJ/mol	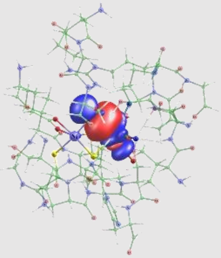	S4(LP)→Fe-C(BD*)-β<br>E_2=164.4 kJ/mol	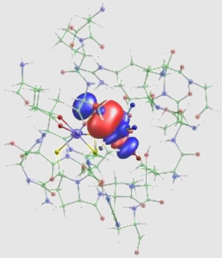	S4(LP)→Fe-C(BD*)<br>E_2=309.2 kJ/mol
12	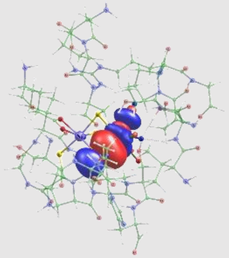	S2(LP)→Fe-C2(BD*)-α<br>E_2=166.1 kJ/mol	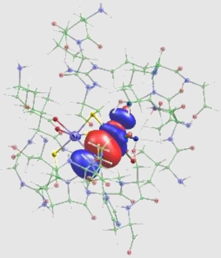	S2(LP)→Fe-C2(BD*)-β<br>E_2=159.0 kJ/mol	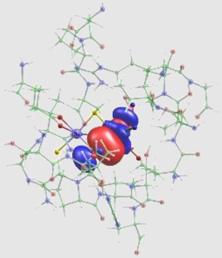	S2(LP)→Fe-C2(BD*)<br>E_2=290.8 kJ/mol

13	-	-	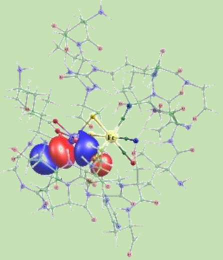	S1(73.7%)-Ni(1.0%)-S2(25.3%)-β (3Cn)	-	-

Table S3. PBE0-optimized large QM energy (kJ/mol) of the broken symmetry (BS) and closed-shell singlet (CS) states relative to the high spin state (HS) and calculated exchange coupling constants J (cm^{-1})

Basis Set	Closed shell	Broken Symmetry	[a]J
**D3(BJ)/def2-SVP (ZORA)***	58.3	-0.8	-62.9

[a]J-values are reported with optimized structures of HS and BS.*ZORA optimization is performed in ORCA program.

Table S4. J-coupling (cm^{-1}) via single point energies on the triplet state (high-spin, HS) optimized structure (BP86/def2-SVP), both with and without dispersion correction.

Basis Set	Broken Symmetry	J
**def2-SVP**	**-4.3**	**-288.6**
**D3(BJ)/def2-SVP**	-4.3	-288.5

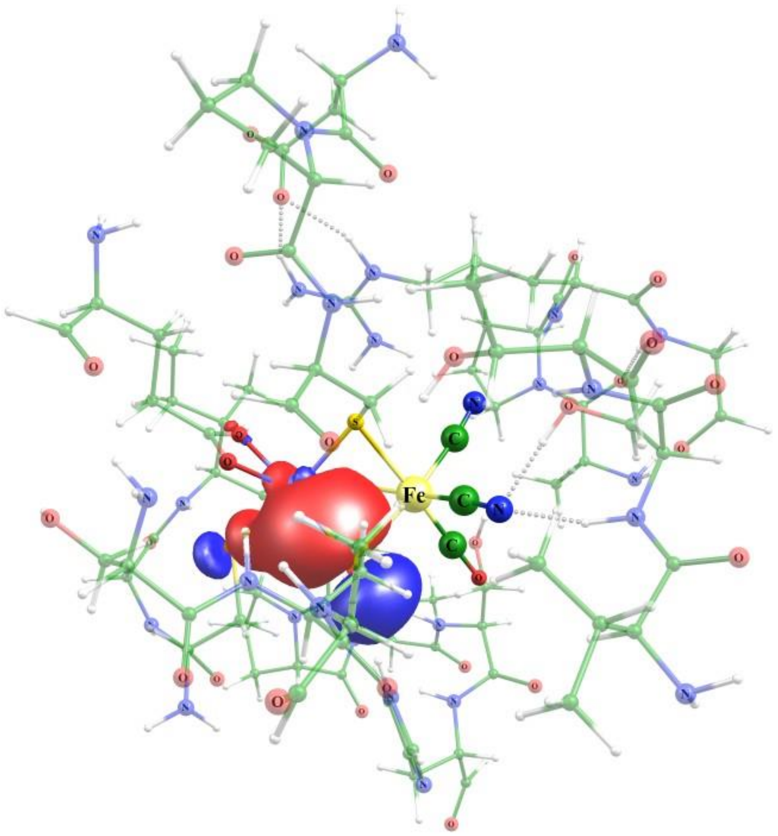

Figure S2. IAO-IBO localization having delocalized three-center (3C) orbital of the broken-symmetry (BS) at PBE0/def2-SVP

Cartesian Coordinates of Optimized Structures

Oxidized [NiFe] hydrogenase active site model

HS_BP86

N	-48.065000000	22.088000000	-34.966000000
C	-47.549047900	20.720028600	-34.765944300
C	-47.141000000	20.185000000	-36.148000000
O	-47.651361800	19.245124900	-36.726265100
C	-48.480951300	19.722924900	-34.071244500
C	-47.780833300	18.377190400	-33.793347700
C	-48.767919900	17.259295600	-33.564240600
O	-49.181050900	16.538358600	-34.526089000
O	-49.195476700	17.021568700	-32.376815600
H	-49.054196200	22.008803000	-35.249973700
H	-48.067304500	22.588467900	-34.068944800
H	-46.343449100	20.811725800	-36.650599900
H	-46.588954200	20.812541400	-34.201226900
H	-49.352566300	19.578224700	-34.738767400
H	-48.871975000	20.165329100	-33.134323000
H	-47.123916800	18.456244200	-32.904379800
H	-47.164509000	18.078989800	-34.662423200
N	-46.173000000	11.189000000	-33.543000000
C	-46.396120700	12.353993600	-32.699589800
C	-45.936659400	12.032468200	-31.253181700
O	-46.282655500	10.965095600	-30.731709900
C	-47.895710800	12.701454300	-32.801868300
S	-48.176402300	14.452017600	-32.376418400
H	-45.165638800	10.991277500	-33.615389000
H	-46.576179500	10.368902300	-33.063911100
H	-45.811711200	13.198239000	-33.120860600
H	-48.484094600	12.010176000	-32.162073400
H	-48.189027100	12.532312900	-33.854834400
N	-45.019738000	12.850910800	-30.632080700
C	-44.853166600	14.293980300	-30.771767500
C	-45.472605400	14.962172500	-29.523317400
O	-44.974712800	14.784644300	-28.407881800
H	-44.718371300	12.491568800	-29.717453700
H	-45.330785000	14.636188500	-31.709564800
H	-43.773978000	14.554955700	-30.802294200
N	-46.620054700	15.654103200	-29.764269600
C	-47.500663800	16.164703000	-28.720184500
C	-48.512749300	15.108391900	-28.242148200
O	-49.002564700	15.136882900	-27.107401700
C	-48.256000000	17.430000000	-29.226000000
C	-47.232000000	18.397000000	-29.828000000
C	-49.003000000	18.127000000	-28.108000000
C	-47.851000000	19.602000000	-30.514000000
H	-47.007329000	15.602474300	-30.719567100
H	-46.872404400	16.415610700	-27.841475300
H	-48.957015500	17.113207000	-30.027150600
H	-46.608153700	17.856668100	-30.568526900
H	-46.525888400	18.718864600	-29.028741300
H	-49.589209200	18.986329800	-28.491867400
H	-49.700399300	17.451717800	-27.574893800
H	-48.297820100	18.527712800	-27.348921700
H	-47.097777300	20.171124300	-31.096790900
H	-48.656004900	19.287472400	-31.205997700
H	-48.290603400	20.322391600	-29.792293300
N	-48.838619700	14.171630200	-29.179785500
C	-49.915827600	13.235876100	-28.975855000
C	-49.461787500	11.775417100	-28.748432900
O	-50.227290700	10.857365300	-29.114232000
C	-50.889471300	13.205128700	-30.180080400
S	-51.353747800	14.851959900	-30.858209700
H	-48.482657400	14.254904500	-30.155372400
H	-50.450667500	13.565713800	-28.055705500

HS_PBE0

N	-48.065000000	22.088000000	-34.966000000
C	-47.549047900	20.720028600	-34.765944300
C	-47.141000000	20.185000000	-36.148000000
O	-47.651361800	19.245124900	-36.726265100
C	-48.480951300	19.722924900	-34.071244500
C	-47.780833300	18.377190400	-33.793347700
C	-48.767919900	17.259295600	-33.564240600
O	-49.181050900	16.538358600	-34.526089000
O	-49.195476700	17.021568700	-32.376815600
H	-49.054196200	22.008803000	-35.249973700
H	-48.067304500	22.588467900	-34.068944800
H	-46.343449100	20.811725800	-36.650599900
H	-46.588954200	20.812541400	-34.201226900
H	-49.352566300	19.578224700	-34.738767400
H	-48.871975000	20.165329100	-33.134323000
H	-47.123916800	18.456244200	-32.904379800
H	-47.164509000	18.078989800	-34.662423200
N	-46.173000000	11.189000000	-33.543000000
C	-46.396120700	12.353993600	-32.699589800
C	-45.936659400	12.032468200	-31.253181700
O	-46.282655500	10.965095600	-30.731709900
C	-47.895710800	12.701454300	-32.801868300
S	-48.176402300	14.452017600	-32.376418400
H	-45.165638800	10.991277500	-33.615389000
H	-46.576179500	10.368902300	-33.063911100
H	-45.811711200	13.198239000	-33.120860600
H	-48.484094600	12.010176000	-32.162073400
H	-48.189027100	12.532312900	-33.854834400
N	-45.019738000	12.850910800	-30.632080700
C	-44.853166600	14.293980300	-30.771767500
C	-45.472605400	14.962172500	-29.523317400
O	-44.974712800	14.784644300	-28.407881800
H	-44.718371300	12.491568800	-29.717453700
H	-45.330785000	14.636188500	-31.709564800
H	-43.773978000	14.554955700	-30.802294200
N	-46.620054700	15.654103200	-29.764269600
C	-47.500663800	16.164703000	-28.720184500
C	-48.512749300	15.108391900	-28.242148200
O	-49.002564700	15.136882900	-27.107401700
C	-48.256000000	17.430000000	-29.226000000
C	-47.232000000	18.397000000	-29.828000000
C	-49.003000000	18.127000000	-28.108000000
C	-47.851000000	19.602000000	-30.514000000
H	-47.007329000	15.602474300	-30.719567100
H	-46.872404400	16.415610700	-27.841475300
H	-48.957015500	17.113207000	-30.027150600
H	-46.608153700	17.856668100	-30.568526900
H	-46.525888400	18.718864600	-29.028741300
H	-49.589209200	18.986329800	-28.491867400
H	-49.700399300	17.451717800	-27.574893800
H	-48.297820100	18.527712800	-27.348921700
H	-47.097777300	20.171124300	-31.096790900
H	-48.656004900	19.287472400	-31.205997700
H	-48.290603400	20.322391600	-29.792293300
N	-48.838619700	14.171630200	-29.179785500
C	-49.915827600	13.235876100	-28.975855000
C	-49.461787500	11.775417100	-28.748432900
O	-50.227290700	10.857365300	-29.114232000
C	-50.889471300	13.205128700	-30.180080400
S	-51.353747800	14.851959900	-30.858209700
H	-48.482657400	14.254904500	-30.155372400
H	-50.450667500	13.565713800	-28.055705500

H	-51.815871000	12.692743800	-29.867394900
H	-50.452134600	12.611613900	-31.005740400
N	-48.272985500	11.467053000	-28.162330800
C	-47.903308000	10.033525300	-28.129404300
C	-48.916517700	9.205966900	-27.316814400
O	-49.208642000	8.045525100	-27.612602700
C	-46.476131200	10.043093300	-27.537001700
C	-46.435251600	11.317696900	-26.676832500
C	-47.270427000	12.322495600	-27.488137100
H	-47.917033600	9.619371300	-29.154560600
H	-45.765221100	10.122065700	-28.383141100
H	-46.251489200	9.115128800	-26.976464900
H	-45.410050500	11.696546600	-26.497521300
H	-46.894437400	11.135218300	-25.682137400
H	-47.759186100	13.095803100	-26.864722600
H	-46.636185400	12.842765500	-28.230945300
N	-49.438853300	9.850536300	-26.227274600
C	-50.451101600	9.258970900	-25.368568100
C	-51.759804900	8.895733400	-26.120535100
O	-52.436511000	7.926756300	-25.777053500
C	-50.730000000	10.208000000	-24.166000000
C	-51.957000000	9.716000000	-23.400000000
C	-49.514000000	10.339000000	-23.256000000
H	-49.228423800	10.843982300	-26.112252400
H	-50.082387100	8.282608000	-24.987576600
H	-50.970609800	11.209279900	-24.595973200
H	-52.105240700	10.320335700	-22.480742600
H	-52.883636800	9.784627300	-23.998720000
H	-51.842202300	8.655328600	-23.097842400
H	-49.707321700	11.066650800	-22.441509700
H	-49.268104400	9.364079600	-22.784308700
H	-48.608259000	10.681169400	-23.797273900
N	-52.139921300	9.765820500	-27.107324300
C	-53.373250700	9.560811900	-27.848102700
C	-53.299322500	8.422064600	-28.894797200
O	-54.321547500	7.897556100	-29.336108100
C	-53.790092500	10.815270800	-28.616378500
O	-53.900531600	11.938666800	-27.747958500
H	-51.462261300	10.437225500	-27.485460400
H	-54.164577800	9.261519800	-27.131900300
H	-54.748230700	10.572630900	-29.124615600
H	-53.031405800	10.994258500	-29.412694000
H	-54.348756300	12.634101300	-28.265161200
N	-52.037592300	8.125458500	-29.337992300
C	-51.804122000	6.965813800	-30.173856500
C	-51.667000000	5.759000000	-29.241000000
O	-50.945952800	4.802790600	-29.439931900
C	-50.590039900	7.098945700	-31.113895400
C	-50.759588300	8.108090400	-32.208228000
N	-51.051674200	7.750781100	-33.509939400
C	-50.676559800	9.500123700	-32.104075100
C	-51.140306100	8.889708100	-34.183679200
N	-50.919115600	9.977747000	-33.370831700
H	-51.230927500	8.500147600	-28.824640400
H	-52.743960700	6.802327000	-30.751750600
H	-52.308431000	5.835739000	-28.313903300
H	-50.415826900	6.102974800	-31.564338200
H	-49.701725600	7.330483300	-30.488362200
H	-50.494942300	10.148725400	-31.239876200
H	-50.928662800	10.973416500	-33.633201000
H	-51.352971800	8.997763300	-35.255682900
N	-58.620000000	12.567000000	-28.890000000
C	-57.473822500	13.418715100	-28.628870000
C	-57.879542100	14.908628400	-28.486242300
O	-59.042609300	15.271709300	-28.743285300
C	-56.443236200	13.201800900	-29.746603100
H	-59.408030400	12.879298700	-28.306985200

H	-51.815871000	12.692743800	-29.867394900
H	-50.452134600	12.611613900	-31.005740400
N	-48.272985500	11.467053000	-28.162330800
C	-47.903308000	10.033525300	-28.129404300
C	-48.916517700	9.205966900	-27.316814400
O	-49.208642000	8.045525100	-27.612602700
C	-46.476131200	10.043093300	-27.537001700
C	-46.435251600	11.317696900	-26.676832500
C	-47.270427000	12.322495600	-27.488137100
H	-47.917033600	9.619371300	-29.154560600
H	-45.765221100	10.122065700	-28.383141100
H	-46.251489200	9.115128800	-26.976464900
H	-45.410050500	11.696546600	-26.497521300
H	-46.894437400	11.135218300	-25.682137400
H	-47.759186100	13.095803100	-26.864722600
H	-46.636185400	12.842765500	-28.230945300
N	-49.438853300	9.850536300	-26.227274600
C	-50.451101600	9.258970900	-25.368568100
C	-51.759804900	8.895733400	-26.120535100
O	-52.436511000	7.926756300	-25.777053500
C	-50.730000000	10.208000000	-24.166000000
C	-51.957000000	9.716000000	-23.400000000
C	-49.514000000	10.339000000	-23.256000000
H	-49.228423800	10.843982300	-26.112252400
H	-50.082387100	8.282608000	-24.987576600
H	-50.970609800	11.209279900	-24.595973200
H	-52.105240700	10.320335700	-22.480742600
H	-52.883636800	9.784627300	-23.998720000
H	-51.842202300	8.655328600	-23.097842400
H	-49.707321700	11.066650800	-22.441509700
H	-49.268104400	9.364079600	-22.784308700
H	-48.608259000	10.681169400	-23.797273900
N	-52.139921300	9.765820500	-27.107324300
C	-53.373250700	9.560811900	-27.848102700
C	-53.299322500	8.422064600	-28.894797200
O	-54.321547500	7.897556100	-29.336108100
C	-53.790092500	10.815270800	-28.616378500
O	-53.900531600	11.938666800	-27.747958500
H	-51.462261300	10.437225500	-27.485460400
H	-54.164577800	9.261519800	-27.131900300
H	-54.748230700	10.572630900	-29.124615600
H	-53.031405800	10.994258500	-29.412694000
H	-54.348756300	12.634101300	-28.265161200
N	-52.037592300	8.125458500	-29.337992300
C	-51.804122000	6.965813800	-30.173856500
C	-51.667000000	5.759000000	-29.241000000
O	-50.945952800	4.802790600	-29.439931900
C	-50.590039900	7.098945700	-31.113895400
C	-50.759588300	8.108090400	-32.208228000
N	-51.051674200	7.750781100	-33.509939400
C	-50.676559800	9.500123700	-32.104075100
C	-51.140306100	8.889708100	-34.183679200
N	-50.919115600	9.977747000	-33.370831700
H	-51.230927500	8.500147600	-28.824640400
H	-52.743960700	6.802327000	-30.751750600
H	-52.308431000	5.835739000	-28.313903300
H	-50.415826900	6.102974800	-31.564338200
H	-49.701725600	7.330483300	-30.488362200
H	-50.494942300	10.148725400	-31.239876200
H	-50.928662800	10.973416500	-33.633201000
H	-51.352971800	8.997763300	-35.255682900
N	-58.620000000	12.567000000	-28.890000000
C	-57.473822500	13.418715100	-28.628870000
C	-57.879542100	14.908628400	-28.486242300
O	-59.042609300	15.271709300	-28.743285300
C	-56.443236200	13.201800900	-29.746603100
H	-59.408030400	12.879298700	-28.306985200

H	-58.929910000	12.735766100	-29.858674100
H	-57.011039300	13.107933700	-27.660195800
H	-55.493975300	13.745831300	-29.582552300
H	-56.246771300	12.116718400	-29.845435500
H	-56.848645600	13.562945100	-30.712356200
N	-56.952949500	15.817983500	-28.057129900
C	-57.363203500	17.216988100	-27.815814500
C	-58.008269300	17.944240200	-29.010479100
O	-58.793161300	18.871282800	-28.805576600
C	-56.062755100	17.887886200	-27.317741500
C	-55.310512400	16.736294300	-26.631813700
C	-55.588413000	15.547951400	-27.566055400
H	-58.149336000	17.243374200	-27.030134200
H	-56.274389300	18.749285500	-26.656732200
H	-55.474429200	18.261037000	-28.182555800
H	-55.739106000	16.540045500	-25.626224600
H	-54.227051300	16.929686000	-26.505742800
H	-54.870928400	15.536123600	-28.416195500
H	-55.535510800	14.569581600	-27.047165600
N	-57.628122700	17.521868700	-30.256067800
C	-58.171653200	18.127671300	-31.461359100
C	-59.295556500	17.294631200	-32.123242400
O	-59.707548300	17.579248100	-33.253976500
C	-57.070971400	18.421037700	-32.497031600
C	-55.943134100	19.313949900	-31.960352800
C	-54.638826800	19.065590900	-32.729799600
N	-53.478074200	19.793636200	-32.228724000
C	-52.661989200	19.375930200	-31.252610300
N	-51.524148300	20.067507500	-31.033633300
N	-52.964841800	18.299848300	-30.479713400
H	-56.872570500	16.825325000	-30.361410500
H	-58.658491100	19.072212500	-31.129364200
H	-57.558818000	18.867423600	-33.385427300
H	-56.659736200	17.447426200	-32.841885400
H	-55.772311300	19.091403500	-30.885655800
H	-56.215847800	20.388802700	-32.021755600
H	-54.758595700	19.367423100	-33.786628900
H	-54.415398300	17.978025800	-32.708055800
H	-53.189239900	20.690640100	-32.683252100
H	-51.023448900	19.936999400	-30.156934700
H	-51.394997500	20.946793100	-31.608716900
H	-52.179442300	17.802704100	-30.053816600
H	-53.737506000	17.668878000	-30.751878700
N	-59.806025300	16.260139100	-31.386937600
C	-60.853346100	15.444507200	-31.935480700
C	-60.497000000	13.991000000	-32.170000000
O	-59.439190900	13.460178400	-31.886171000
H	-59.437718400	16.032673100	-30.447071700
H	-61.154369200	15.896976700	-32.910099200
H	-61.776364000	15.446076500	-31.303956000
H	-61.343993400	13.391718800	-32.627273600
N	-56.890000000	9.950000000	-38.249000000
C	-56.357246500	11.245252300	-37.842525100
C	-57.513662500	12.146755700	-37.303704600
O	-58.641375100	12.038981500	-37.795861700
C	-55.671316100	11.931931200	-39.066860400
C	-54.937796600	13.218936300	-38.673948000
C	-54.715771700	10.966370300	-39.786742100
H	-57.125384000	9.391829200	-37.418043800
H	-57.793254000	10.133338800	-38.715303500
H	-55.591740800	11.094698400	-37.049157100
H	-56.498740700	12.193141200	-39.767642100
H	-54.539282800	13.720125000	-39.578755300
H	-55.588248900	13.942940000	-38.148854300
H	-54.079924400	12.991616400	-38.007959900
H	-54.367194800	11.412326700	-40.742316400
H	-53.820339500	10.759442100	-39.163833500

H	-58.929910000	12.735766100	-29.858674100
H	-57.011039300	13.107933700	-27.660195800
H	-55.493975300	13.745831300	-29.582552300
H	-56.246771300	12.116718400	-29.845435500
H	-56.848645600	13.562945100	-30.712356200
N	-56.952949500	15.817983500	-28.057129900
C	-57.363203500	17.216988100	-27.815814500
C	-58.008269300	17.944240200	-29.010479100
O	-58.793161300	18.871282800	-28.805576600
C	-56.062755100	17.887886200	-27.317741500
C	-55.310512400	16.736294300	-26.631813700
C	-55.588413000	15.547951400	-27.566055400
H	-58.149336000	17.243374200	-27.030134200
H	-56.274389300	18.749285500	-26.656732200
H	-55.474429200	18.261037000	-28.182555800
H	-55.739106000	16.540045500	-25.626224600
H	-54.227051300	16.929686000	-26.505742800
H	-54.870928400	15.536123600	-28.416195500
H	-55.535510800	14.569581600	-27.047165600
N	-57.628122700	17.521868700	-30.256067800
C	-58.171653200	18.127671300	-31.461359100
C	-59.295556500	17.294631200	-32.123242400
O	-59.707548300	17.579248100	-33.253976500
C	-57.070971400	18.421037700	-32.497031600
C	-55.943134100	19.313949900	-31.960352800
C	-54.638826800	19.065590900	-32.729799600
N	-53.478074200	19.793636200	-32.228724000
C	-52.661989200	19.375930200	-31.252610300
N	-51.524148300	20.067507500	-31.033633300
N	-52.964841800	18.299848300	-30.479713400
H	-56.872570500	16.825325000	-30.361410500
H	-58.658491100	19.072212500	-31.129364200
H	-57.558818000	18.867423600	-33.385427300
H	-56.659736200	17.447426200	-32.841885400
H	-55.772311300	19.091403500	-30.885655800
H	-56.215847800	20.388802700	-32.021755600
H	-54.758595700	19.367423100	-33.786628900
H	-54.415398300	17.978025800	-32.708055800
H	-53.189239900	20.690640100	-32.683252100
H	-51.023448900	19.936999400	-30.156934700
H	-51.394997500	20.946793100	-31.608716900
H	-52.179442300	17.802704100	-30.053816600
H	-53.737506000	17.668878000	-30.751878700
N	-59.806025300	16.260139100	-31.386937600
C	-60.853346100	15.444507200	-31.935480700
C	-60.497000000	13.991000000	-32.170000000
O	-59.439190900	13.460178400	-31.886171000
H	-59.437718400	16.032673100	-30.447071700
H	-61.154369200	15.896976700	-32.910099200
H	-61.776364000	15.446076500	-31.303956000
H	-61.343993400	13.391718800	-32.627273600
N	-56.890000000	9.950000000	-38.249000000
C	-56.357246500	11.245252300	-37.842525100
C	-57.513662500	12.146755700	-37.303704600
O	-58.641375100	12.038981500	-37.795861700
C	-55.671316100	11.931931200	-39.066860400
C	-54.937796600	13.218936300	-38.673948000
C	-54.715771700	10.966370300	-39.786742100
H	-57.125384000	9.391829200	-37.418043800
H	-57.793254000	10.133338800	-38.715303500
H	-55.591740800	11.094698400	-37.049157100
H	-56.498740700	12.193141200	-39.767642100
H	-54.539282800	13.720125000	-39.578755300
H	-55.588248900	13.942940000	-38.148854300
H	-54.079924400	12.991616400	-38.007959900
H	-54.367194800	11.412326700	-40.742316400
H	-53.820339500	10.759442100	-39.163833500

H	-55.216770800	10.005272000	-40.005459500	H	-55.216770800	10.005272000	-40.005459500
N	-57.222490700	13.049342400	-36.312981300	N	-57.222490700	13.049342400	-36.312981300
C	-58.186408000	14.102496400	-35.973510500	C	-58.186408000	14.102496400	-35.973510500
C	-58.162218000	15.184180300	-37.072595600	C	-58.162218000	15.184180300	-37.072595600
O	-59.082611700	15.337355800	-37.882205900	O	-59.082611700	15.337355800	-37.882205900
C	-57.978720800	14.692003200	-34.566684300	C	-57.978720800	14.692003200	-34.566684300
O	-56.805536600	15.483256400	-34.425858800	O	-56.805536600	15.483256400	-34.425858800
H	-56.246772900	13.168106100	-35.985433200	H	-56.246772900	13.168106100	-35.985433200
H	-59.196978000	13.652265600	-36.033691200	H	-59.196978000	13.652265600	-36.033691200
H	-58.004607100	13.865922700	-33.823141300	H	-58.004607100	13.865922700	-33.823141300
H	-58.831894700	15.370415200	-34.359706900	H	-58.831894700	15.370415200	-34.359706900
H	-56.025986300	14.875608200	-34.398524100	H	-56.025986300	14.875608200	-34.398524100
N	-56.984332000	15.877755800	-37.173758000	N	-56.984332000	15.877755800	-37.173758000
C	-56.822952200	16.762466800	-38.299536500	C	-56.822952200	16.762466800	-38.299536500
C	-57.299000000	16.002000000	-39.542000000	C	-57.299000000	16.002000000	-39.542000000
O	-57.610923600	16.545928300	-40.585562000	O	-57.610923600	16.545928300	-40.585562000
C	-55.333835700	17.129053900	-38.562608100	C	-55.333835700	17.129053900	-38.562608100
O	-54.614069800	17.271874000	-37.324706700	O	-54.614069800	17.271874000	-37.324706700
C	-55.236110400	18.396879100	-39.407409000	C	-55.236110400	18.396879100	-39.407409000
H	-57.200021100	14.883511000	-39.461161900	H	-57.200021100	14.883511000	-39.461161900
H	-56.401612700	15.966680900	-36.327966400	H	-56.401612700	15.966680900	-36.327966400
H	-57.428224500	17.693808400	-38.202241400	H	-57.428224500	17.693808400	-38.202241400
H	-54.874665400	16.281083600	-39.119144800	H	-54.874665400	16.281083600	-39.119144800
H	-53.821721600	16.691969700	-37.398569700	H	-53.821721600	16.691969700	-37.398569700
H	-54.188999700	18.585333200	-39.717555300	H	-54.188999700	18.585333200	-39.717555300
H	-55.870599300	18.303315400	-40.311198300	H	-55.870599300	18.303315400	-40.311198300
H	-55.600054200	19.268306300	-38.824513300	H	-55.600054200	19.268306300	-38.824513300
N	-55.492000000	24.131000000	-34.333000000	N	-55.492000000	24.131000000	-34.333000000
C	-54.084762300	23.814450300	-34.069409100	C	-54.084762300	23.814450300	-34.069409100
C	-53.862128900	22.516608500	-34.864593800	C	-53.862128900	22.516608500	-34.864593800
O	-54.485494300	21.490828300	-34.551999000	O	-54.485494300	21.490828300	-34.551999000
C	-53.757681100	23.663453400	-32.564610300	C	-53.757681100	23.663453400	-32.564610300
C	-52.309286700	23.230736000	-32.245754800	C	-52.309286700	23.230736000	-32.245754800
O	-51.952746400	22.085800700	-32.711313100	O	-51.952746400	22.085800700	-32.711313100
O	-51.597611400	23.989122500	-31.554956700	O	-51.597611400	23.989122500	-31.554956700
H	-56.059064300	23.317821500	-34.052221200	H	-56.059064300	23.317821500	-34.052221200
H	-55.794507600	24.923783000	-33.752150400	H	-55.794507600	24.923783000	-33.752150400
H	-53.461591700	24.635260600	-34.479451800	H	-53.461591700	24.635260600	-34.479451800
H	-54.436308500	22.888917300	-32.145032700	H	-54.436308500	22.888917300	-32.145032700
H	-53.974826100	24.626020000	-32.059067300	H	-53.974826100	24.626020000	-32.059067300
N	-53.086412800	22.559546100	-35.985377200	N	-53.086412800	22.559546100	-35.985377200
C	-53.032285100	21.382626900	-36.860102000	C	-53.032285100	21.382626900	-36.860102000
C	-52.079249800	20.316122900	-36.281664400	C	-52.079249800	20.316122900	-36.281664400
O	-50.933552900	20.600337700	-35.921771200	O	-50.933552900	20.600337700	-35.921771200
C	-52.492874500	21.963696800	-38.188868900	C	-52.492874500	21.963696800	-38.188868900
C	-51.586762600	23.120695000	-37.732638200	C	-51.586762600	23.120695000	-37.732638200
C	-52.328723900	23.705684600	-36.517238100	C	-52.328723900	23.705684600	-36.517238100
H	-54.051018200	20.955358400	-36.957911400	H	-54.051018200	20.955358400	-36.957911400
H	-53.348162800	22.341511900	-38.787044100	H	-53.348162800	22.341511900	-38.787044100
H	-51.964180700	21.207339600	-38.801472600	H	-51.964180700	21.207339600	-38.801472600
H	-51.417324800	23.877328900	-38.523934000	H	-51.417324800	23.877328900	-38.523934000
H	-50.603181800	22.722666700	-37.417181300	H	-50.603181800	22.722666700	-37.417181300
H	-51.630644900	24.110828600	-35.757666300	H	-51.630644900	24.110828600	-35.757666300
H	-53.024928100	24.520227300	-36.818250400	H	-53.024928100	24.520227300	-36.818250400
N	-52.591613700	19.047027700	-36.242768800	N	-52.591613700	19.047027700	-36.242768800
C	-51.811913300	17.879120600	-35.814696700	C	-51.811913300	17.879120600	-35.814696700
C	-51.292000000	17.314000000	-37.146000000	C	-51.292000000	17.314000000	-37.146000000
O	-51.869039300	16.429810300	-37.770590600	O	-51.869039300	16.429810300	-37.770590600
C	-52.657716500	16.867134900	-35.014397800	C	-52.657716500	16.867134900	-35.014397800
S	-52.085277600	16.504877700	-33.304959000	S	-52.085277600	16.504877700	-33.304959000
H	-50.379949200	17.818718600	-37.553285900	H	-50.379949200	17.818718600	-37.553285900
H	-53.523100400	18.832512600	-36.639483400	H	-53.523100400	18.832512600	-36.639483400
H	-50.947463400	18.258970000	-35.238495300	H	-50.947463400	18.258970000	-35.238495300
H	-52.797808700	15.928131800	-35.579939500	H	-52.797808700	15.928131800	-35.579939500
H	-53.678759500	17.265821100	-34.854257800	H	-53.678759500	17.265821100	-34.854257800
N	-48.129000000	16.435000000	-37.652000000	N	-48.129000000	16.435000000	-37.652000000
C	-47.269200900	15.256475400	-37.484212400	C	-47.269200900	15.256475400	-37.484212400

C	-48.106829200	13.960187700	-37.372193800
O	-47.737528300	12.876146300	-37.819840400
C	-46.415814800	15.373256000	-36.214183900
O	-45.630232000	16.563168600	-36.306984300
H	-47.658670000	17.276263700	-37.286834000
H	-48.272193100	16.611793500	-38.653916700
H	-46.575524600	15.084128400	-38.339942000
H	-45.780626500	14.459244800	-36.147384800
H	-47.086703400	15.400259600	-35.325932100
H	-45.211660100	16.702704500	-35.439375500
N	-49.294335600	14.148068700	-36.708026300
C	-50.268395400	13.087327500	-36.568444800
C	-50.949795400	12.681324800	-37.916291100
O	-51.522529200	11.596582500	-38.020783400
C	-51.412604800	13.472909600	-35.624070300
S	-51.040826100	13.429399300	-33.817785500
H	-49.478275200	15.107115200	-36.381973200
H	-49.764157000	12.156479300	-36.231051800
H	-52.239422500	12.747308200	-35.751380500
H	-51.812355800	14.462648400	-35.908154200
N	-50.913295900	13.634198100	-38.892041800
C	-51.534209300	13.470007700	-40.199545800
C	-50.531000000	12.863000000	-41.188000000
O	-50.472221100	13.148649300	-42.367357900
C	-52.144797900	14.763039500	-40.735549600
H	-49.833133900	12.106564200	-40.719770000
H	-50.454072300	14.523078000	-38.674512400
H	-52.315707200	12.683600400	-40.069091400
H	-52.584372500	14.580416000	-41.733777900
H	-52.923715900	15.146389700	-40.052284200
H	-51.370391100	15.548238500	-40.854150200
Fe	-52.862635600	14.383769000	-32.577669900
C	-53.970065000	14.089556000	-34.048132600
N	-54.653304500	13.805906100	-34.974412300
C	-54.195629300	15.323756700	-31.641626800
N	-54.945308600	15.994976400	-31.015099500
C	-53.365450700	12.821482000	-31.991990000
O	-53.747837600	11.770316200	-31.672501800
Ni	-50.164299800	15.336609800	-32.985439600

BS_BP86

N	-48.065000000	22.088000000	-34.966000000
C	-47.563129200	20.716020600	-34.768551700
C	-47.141000000	20.185000000	-36.148000000
O	-47.643870000	19.243940800	-36.729867700
C	-48.511095500	19.721288300	-34.091718000
C	-47.813431700	18.374211700	-33.812127500
C	-48.790838500	17.262032700	-33.555386500
O	-49.217448600	16.515347100	-34.495960400
O	-49.208360700	17.024983400	-32.363171200
H	-49.046314200	22.020601500	-35.278722600
H	-48.088424400	22.577963300	-34.063470200
H	-46.341282400	20.815291000	-36.642321000
H	-46.610232600	20.797489300	-34.189914700
H	-49.373851500	19.581981100	-34.772540500
H	-48.911753900	20.158360800	-33.156205600
H	-47.144884900	18.459179900	-32.931966100
H	-47.209396800	18.066380900	-34.686610400
N	-46.173000000	11.189000000	-33.543000000
C	-46.398598000	12.358884500	-32.710412300
C	-45.962832000	12.041212800	-31.255565100
O	-46.332674000	10.983246800	-30.732197200
C	-47.895311000	12.714808900	-32.837070500
S	-48.160426700	14.465714600	-32.415570500
H	-45.166364100	10.989517700	-33.615621400
H	-46.582403400	10.371508200	-33.065234500
H	-45.802275500	13.196791500	-33.127663300
H	-48.496868300	12.029315400	-32.202711300

C	-48.106829200	13.960187700	-37.372193800
O	-47.737528300	12.876146300	-37.819840400
C	-46.415814800	15.373256000	-36.214183900
O	-45.630232000	16.563168600	-36.306984300
H	-47.658670000	17.276263700	-37.286834000
H	-48.272193100	16.611793500	-38.653916700
H	-46.575524600	15.084128400	-38.339942000
H	-45.780626500	14.459244800	-36.147384800
H	-47.086703400	15.400259600	-35.325932100
H	-45.211660100	16.702704500	-35.439375500
N	-49.294335600	14.148068700	-36.708026300
C	-50.268395400	13.087327500	-36.568444800
C	-50.949795400	12.681324800	-37.916291100
O	-51.522529200	11.596582500	-38.020783400
C	-51.412604800	13.472909600	-35.624070300
S	-51.040826100	13.429399300	-33.817785500
H	-49.478275200	15.107115200	-36.381973200
H	-49.764157000	12.156479300	-36.231051800
H	-52.239422500	12.747308200	-35.751380500
H	-51.812355800	14.462648400	-35.908154200
N	-50.913295900	13.634198100	-38.892041800
C	-51.534209300	13.470007700	-40.199545800
C	-50.531000000	12.863000000	-41.188000000
O	-50.472221100	13.148649300	-42.367357900
C	-52.144797900	14.763039500	-40.735549600
H	-49.833133900	12.106564200	-40.719770000
H	-50.454072300	14.523078000	-38.674512400
H	-52.315707200	12.683600400	-40.069091400
H	-52.584372500	14.580416000	-41.733777900
H	-52.923715900	15.146389700	-40.052284200
H	-51.370391100	15.548238500	-40.854150200
Fe	-52.862635600	14.383769000	-32.577669900
C	-53.970065000	14.089556000	-34.048132600
N	-54.653304500	13.805906100	-34.974412300
C	-54.195629300	15.323756700	-31.641626800
N	-54.945308600	15.994976400	-31.015099500
C	-53.365450700	12.821482000	-31.991990000
O	-53.747837600	11.770316200	-31.672501800
Ni	-50.164299800	15.336609800	-32.985439600

BS_PBE0

N	-48.065000000	22.088000000	-34.966000000
C	-47.549047900	20.720028600	-34.765944300
C	-47.141000000	20.185000000	-36.148000000
O	-47.651361800	19.245124900	-36.726265100
C	-48.480951300	19.722924900	-34.071244500
C	-47.780833300	18.377190400	-33.793347700
C	-48.767919900	17.259295600	-33.564240600
O	-49.181050900	16.538358600	-34.526089000
O	-49.195476700	17.021568700	-32.376815600
H	-49.054196200	22.008803000	-35.249973700
H	-48.067304500	22.588467900	-34.068944800
H	-46.343449100	20.811725800	-36.650599900
H	-46.588954200	20.812541400	-34.201226900
H	-49.352566300	19.578224700	-34.738767400
H	-48.871975000	20.165329100	-33.134323000
H	-47.123916800	18.456244200	-32.904379800
H	-47.164509000	18.078989800	-34.662423200
N	-46.173000000	11.189000000	-33.543000000
C	-46.396120700	12.353993600	-32.699589800
C	-45.936659400	12.032468200	-31.253181700
O	-46.282655500	10.965095600	-30.731709900
C	-47.895710800	12.701454300	-32.801868300
S	-48.176402300	14.452017600	-32.376418400
H	-45.165638800	10.991277500	-33.615389000
H	-46.576179500	10.368902300	-33.063911100
H	-45.811711200	13.198239000	-33.120860600
H	-48.484094600	12.010176000	-32.162073400

H	-48.174891600	12.546433500	-33.893939900
N	-45.036709400	12.846402700	-30.629687100
C	-44.850796300	14.287920100	-30.764413400
C	-45.478077100	14.958576500	-29.520933300
O	-44.989117900	14.780435900	-28.401924300
H	-44.749428900	12.482810700	-29.712138300
H	-45.311940300	14.637853900	-31.707620900
H	-43.768372500	14.535595300	-30.780569600
N	-46.625848800	15.648628100	-29.768865700
C	-47.519461000	16.150379100	-28.731518000
C	-48.554296400	15.096737500	-28.292868200
O	-49.084059900	15.124748500	-27.176692200
C	-48.256000000	17.430000000	-29.226000000
C	-47.232000000	18.397000000	-29.828000000
C	-49.003000000	18.127000000	-28.108000000
C	-47.851000000	19.602000000	-30.514000000
H	-46.999898200	15.607270100	-30.729174500
H	-46.903390200	16.377039500	-27.837718600
H	-48.960391400	17.125104000	-30.028909100
H	-46.607717100	17.856472000	-30.568351200
H	-46.524960400	18.718970100	-29.029596200
H	-49.585595200	18.987780800	-28.493334100
H	-49.702325500	17.451789400	-27.577447100
H	-48.297883700	18.524649300	-27.347163000
H	-47.098920200	20.165327000	-31.103940100
H	-48.664601300	19.291917700	-31.198069400
H	-48.282099000	20.326794800	-29.791885000
N	-48.850423800	14.161498700	-29.242864600
C	-49.917327400	13.208149300	-29.069301900
C	-49.446363100	11.755229100	-28.817010600
O	-50.186128800	10.824698500	-29.201599400
C	-50.866689900	13.156890600	-30.295870300
S	-51.323191300	14.791736800	-31.007071600
H	-48.459007100	14.244234800	-30.203495500
H	-50.484559900	13.531136700	-28.166396400
H	-51.810192600	12.668386900	-29.994321200
H	-50.431653500	12.542876600	-31.107986500
N	-48.275486000	11.470801700	-28.183906400
C	-47.885087200	10.043564800	-28.122038900
C	-48.886414100	9.211700500	-27.298866600
O	-49.135944000	8.035024400	-27.562078400
C	-46.464128800	10.085301400	-27.517930200
C	-46.459104000	11.364888900	-26.664933700
C	-47.303268900	12.348335700	-27.492938400
H	-47.885298700	9.611377100	-29.139483700
H	-45.746360100	10.172802100	-28.357580100
H	-46.226549900	9.165080300	-26.950152500
H	-45.444441600	11.766102700	-26.474834000
H	-46.927889000	11.177873800	-25.675816500
H	-47.819103700	13.111573800	-26.878955200
H	-46.666934600	12.881023400	-28.224803900
N	-49.444393300	9.873472500	-26.235815200
C	-50.458595000	9.276142900	-25.383482800
C	-51.769248600	8.931398400	-26.144506500
O	-52.467698700	7.978767000	-25.799754700
C	-50.730000000	10.208000000	-24.166000000
C	-51.957000000	9.716000000	-23.400000000
C	-49.514000000	10.339000000	-23.256000000
H	-49.268124800	10.876268500	-26.147107000
H	-50.092323700	8.291863400	-25.021814900
H	-50.971838300	11.214060800	-24.585405700
H	-52.104622600	10.320993300	-22.481064400
H	-52.884061300	9.781692700	-23.998074200
H	-51.841412300	8.655694700	-23.096393500
H	-49.710893200	11.062517500	-22.438827200
H	-49.263632500	9.363315300	-22.788032500
H	-48.610483800	10.686620600	-23.797390400

H	-48.189027100	12.532312900	-33.854834400
N	-45.019738000	12.850910800	-30.632080700
C	-44.853166600	14.293980300	-30.771767500
C	-45.472605400	14.962172500	-29.523317400
O	-44.974712800	14.784644300	-28.407881800
H	-44.718371300	12.491568800	-29.717453700
H	-45.330785000	14.636188500	-31.709564800
H	-43.773978000	14.554955700	-30.802294200
N	-46.620054700	15.654103200	-29.764269600
C	-47.500663800	16.164703000	-28.720184500
C	-48.512749300	15.108391900	-28.242148200
O	-49.002564700	15.136882900	-27.107401700
C	-48.256000000	17.430000000	-29.226000000
C	-47.232000000	18.397000000	-29.828000000
C	-49.003000000	18.127000000	-28.108000000
C	-47.851000000	19.602000000	-30.514000000
H	-47.007329000	15.602474300	-30.719567100
H	-46.872404400	16.415610700	-27.841475300
H	-48.957015500	17.113207000	-30.027150600
H	-46.608153700	17.856668100	-30.568526900
H	-46.525888400	18.718864600	-29.028741300
H	-49.589209200	18.986329800	-28.491867400
H	-49.700399300	17.451717800	-27.574893800
H	-48.297820100	18.527712800	-27.348921700
H	-47.097777300	20.171124300	-31.096790900
H	-48.656004900	19.287472400	-31.205997700
H	-48.290603400	20.322391600	-29.792293300
N	-48.838619700	14.171630200	-29.179785500
C	-49.915827600	13.235876100	-28.975855000
C	-49.461787500	11.775417100	-28.748432900
O	-50.227290700	10.857365300	-29.114232000
C	-50.889471300	13.205128700	-30.180080400
S	-51.353747800	14.851959900	-30.858209700
H	-48.482657400	14.254904500	-30.155372400
H	-50.450667500	13.565713800	-28.055705500
H	-51.815871000	12.692743800	-29.867394900
H	-50.452134600	12.611613900	-31.005740400
N	-48.272985500	11.467053000	-28.162330800
C	-47.903308000	10.033525300	-28.129404300
C	-48.916517700	9.205966900	-27.316814400
O	-49.208642000	8.045525100	-27.612602700
C	-46.476131200	10.043093300	-27.537001700
C	-46.435251600	11.317696900	-26.676832500
C	-47.270427000	12.322495600	-27.488137100
H	-47.917033600	9.619371300	-29.154560600
H	-45.765221100	10.122065700	-28.383141100
H	-46.251489200	9.115128800	-26.976464900
H	-45.410050500	11.696546600	-26.497521300
H	-46.894437400	11.135218300	-25.682137400
H	-47.759186100	13.095803100	-26.864722600
H	-46.636185400	12.842765500	-28.230945300
N	-49.438853300	9.850536300	-26.227274600
C	-50.451101600	9.258970900	-25.368568100
C	-51.759804900	8.895733400	-26.120535100
O	-52.436511000	7.926756300	-25.777053500
C	-50.730000000	10.208000000	-24.166000000
C	-51.957000000	9.716000000	-23.400000000
C	-49.514000000	10.339000000	-23.256000000
H	-49.228423800	10.843982300	-26.112252400
H	-50.082387100	8.282608000	-24.987576600
H	-50.970609800	11.209279900	-24.595973200
H	-52.105240700	10.320335700	-22.480742600
H	-52.883636800	9.784627300	-23.998720000
H	-51.842202300	8.655328600	-23.097842400
H	-49.707321700	11.066650800	-22.441509700
H	-49.268104400	9.364079600	-22.784308700
H	-48.608259000	10.681169400	-23.797273900

N	-52.121276000	9.795201000	-27.147825200
C	-53.350081400	9.604887800	-27.902234200
C	-53.300456200	8.422578900	-28.901915400
O	-54.330583100	7.873496400	-29.290836100
C	-53.703397800	10.839281300	-28.734726400
O	-53.763736800	12.014472600	-27.929828800
H	-51.429939200	10.455438100	-27.517546500
H	-54.166140300	9.366123700	-27.191322900
H	-54.669733200	10.618119300	-29.235580200
H	-52.935336100	10.943244600	-29.536087700
H	-54.298721200	12.658520900	-28.431156000
N	-52.050237100	8.126853200	-29.375856300
C	-51.825387300	6.949989000	-30.191527200
C	-51.667000000	5.759000000	-29.241000000
O	-50.962702600	4.792034300	-29.449775400
C	-50.629922800	7.073520600	-31.156204700
C	-50.807663600	8.078534700	-32.255183500
N	-51.183161200	7.720180100	-33.536326400
C	-50.647872700	9.462552500	-32.176879500
C	-51.248619100	8.852372800	-34.223008700
N	-50.929924800	9.936893300	-33.439635900
H	-51.234629500	8.540724300	-28.910437700
H	-52.772884000	6.771575700	-30.751930600
H	-52.274305600	5.858394500	-28.293185100
H	-50.468849400	6.073052000	-31.601719100
H	-49.727215300	7.303453000	-30.550976400
H	-50.390308900	10.112063800	-31.333382800
H	-50.909474500	10.926181000	-33.715275800
H	-51.505456200	8.957536800	-35.285563000
N	-58.620000000	12.567000000	-28.890000000
C	-57.485136000	13.431133500	-28.621957300
C	-57.905712000	14.916605600	-28.476753600
O	-59.070346500	15.270407000	-28.739128600
C	-56.448784800	13.228609000	-29.736859200
H	-59.412107500	12.863221600	-28.304189000
H	-58.932198700	12.738626700	-29.857412600
H	-57.023232900	13.123989500	-27.651733000
H	-55.510660500	13.792396900	-29.574027100
H	-56.235080400	12.146501300	-29.833940400
H	-56.859129300	13.579177900	-30.704530500
N	-56.989038800	15.832295100	-28.039815200
C	-57.411752900	17.227512400	-27.797545500
C	-58.051134600	17.953082500	-28.996659500
O	-58.844637800	18.873884500	-28.798585800
C	-56.120721000	17.906464100	-27.285545000
C	-55.363602800	16.757269700	-26.600663500
C	-55.623832000	15.572837600	-27.544557200
H	-58.205447900	17.245390700	-27.019364200
H	-56.344490900	18.762275000	-26.621325300
H	-55.529274700	18.289590000	-28.143799900
H	-55.798095300	16.551073400	-25.599584300
H	-54.282903800	16.959573000	-26.464860500
H	-54.903909000	15.576922300	-28.392595100
H	-55.561197700	14.590149500	-27.035356300
N	-57.656223400	17.534475900	-30.239151600
C	-58.192716300	18.134715900	-31.450656900
C	-59.321089000	17.305493500	-32.111611000
O	-59.743861600	17.600853200	-33.235255400
C	-57.085009700	18.408911800	-32.483761500
C	-55.955441400	19.306156200	-31.957923700
C	-54.650338100	19.037770500	-32.719612600
N	-53.490974500	19.784527600	-32.244468100
C	-52.672048400	19.408939600	-31.253444500
N	-51.540175000	20.118242400	-31.054220900
N	-52.964108200	18.351967200	-30.451676500
H	-56.894906600	16.843578200	-30.334359500
H	-58.673115000	19.086097000	-31.129594300

N	-52.139921300	9.765820500	-27.107324300
C	-53.373250700	9.560811900	-27.848102700
C	-53.299322500	8.422064600	-28.894797200
O	-54.321547500	7.897556100	-29.336108100
C	-53.790092500	10.815270800	-28.616378500
O	-53.900531600	11.938666800	-27.747958500
H	-51.462261300	10.437225500	-27.485460400
H	-54.164577800	9.261519800	-27.131900300
H	-54.748230700	10.572630900	-29.124615600
H	-53.031405800	10.994258500	-29.412694000
H	-54.348756300	12.634101300	-28.265161200
N	-52.037592300	8.125458500	-29.337992300
C	-51.804122000	6.965813800	-30.173856500
C	-51.667000000	5.759000000	-29.241000000
O	-50.945952800	4.802790600	-29.439931900
C	-50.590039900	7.098945700	-31.113895400
C	-50.759588300	8.108090400	-32.208228000
N	-51.051674200	7.750781100	-33.509939400
C	-50.676559800	9.500123700	-32.104075100
C	-51.140306100	8.889708100	-34.183679200
N	-50.919115600	9.977747000	-33.370831700
H	-51.230927500	8.500147600	-28.824640400
H	-52.743960700	6.802327000	-30.751750600
H	-52.308431000	5.835739000	-28.313903300
H	-50.415826900	6.102974800	-31.564338200
H	-49.701725600	7.330483300	-30.488362200
H	-50.494942300	10.148725400	-31.239876200
H	-50.928662800	10.973416500	-33.633201000
H	-51.352971800	8.997763300	-35.255682900
N	-58.620000000	12.567000000	-28.890000000
C	-57.473822500	13.418715100	-28.628870000
C	-57.879542100	14.908628400	-28.486242300
O	-59.042609300	15.271709300	-28.743285300
C	-56.443236200	13.201800900	-29.746603100
H	-59.408030400	12.879298700	-28.306985200
H	-58.929910000	12.735766100	-29.858674100
H	-57.011039300	13.107933700	-27.660195800
H	-55.493975300	13.745831300	-29.582552300
H	-56.246771300	12.116718400	-29.845435500
H	-56.848645600	13.562945100	-30.712356200
N	-56.952949500	15.817983500	-28.057129900
C	-57.363203500	17.216988100	-27.815814500
C	-58.008269300	17.944240200	-29.010479100
O	-58.793161300	18.871282800	-28.805576600
C	-56.062755100	17.887886200	-27.317741500
C	-55.310512400	16.736294300	-26.631813700
C	-55.588413000	15.547951400	-27.566055400
H	-58.149336000	17.243374200	-27.030134200
H	-56.274389300	18.749285500	-26.656732200
H	-55.474429200	18.261037000	-28.182555800
H	-55.739106000	16.540045500	-25.626224600
H	-54.227051300	16.929686000	-26.505742800
H	-54.870928400	15.536123600	-28.416195500
H	-55.535510800	14.569581600	-27.047165600
N	-57.628122700	17.521868700	-30.256067800
C	-58.171653200	18.127671300	-31.461359100
C	-59.295556500	17.294631200	-32.123242400
O	-59.707548300	17.579248100	-33.253976500
C	-57.070971400	18.421037700	-32.497031600
C	-55.943134100	19.313949900	-31.960352800
C	-54.638826800	19.065590900	-32.729799600
N	-53.478074200	19.793636200	-32.228724000
C	-52.661989200	19.375930200	-31.252610300
N	-51.524148300	20.067507500	-31.033633300
N	-52.964841800	18.299848300	-30.479713400
H	-56.872570500	16.825325000	-30.361410500
H	-58.658491100	19.072212500	-31.129364200

H	-57.565845200	18.840737800	-33.383000700	H	-57.558818000	18.867423600	-33.385427300
H	-56.677161000	17.427051300	-32.807276400	H	-56.659736200	17.447426200	-32.841885400
H	-55.789255400	19.102972200	-30.878797800	H	-55.772311300	19.091403500	-30.885655800
H	-56.222762700	20.380523500	-32.042925700	H	-56.215847800	20.388802700	-32.021755600
H	-54.774539500	19.308111400	-33.784607700	H	-54.758595700	19.367423100	-33.786628900
H	-54.423515200	17.952419400	-32.663403600	H	-54.415398300	17.978025800	-32.708055800
H	-53.231765900	20.677140600	-32.717804400	H	-53.189239900	20.690640100	-32.683252100
H	-51.085046500	20.058097800	-30.144632900	H	-51.023448900	19.936999400	-30.156934700
H	-51.444925900	21.006069600	-31.635840600	H	-51.394997500	20.946793100	-31.608716900
H	-52.175488600	17.895704000	-29.990208700	H	-52.179442300	17.802704100	-30.053816600
H	-53.728064900	17.698552700	-30.699054800	H	-53.737506000	17.668878000	-30.751878700
N	-59.820145500	16.261340900	-31.380801300	N	-59.806025300	16.260139100	-31.386937600
C	-60.863948400	15.441235800	-31.928504200	C	-60.853346100	15.444507200	-31.935480700
C	-60.497000000	13.991000000	-32.170000000	C	-60.497000000	13.991000000	-32.170000000
O	-59.447519900	13.459675900	-31.857978000	O	-59.439190900	13.460178400	-31.886171000
H	-59.448649000	16.031502900	-30.442869600	H	-59.437718400	16.032673100	-30.447071700
H	-61.178874300	15.898439500	-32.895861700	H	-61.154369200	15.896976700	-32.910099200
H	-61.781877600	15.428470000	-31.289146000	H	-61.776364000	15.446076500	-31.303956000
H	-61.329494500	13.393365200	-32.655009800	H	-61.343993400	13.391718800	-32.627273600
N	-56.890000000	9.950000000	-38.249000000	N	-56.890000000	9.950000000	-38.249000000
C	-56.419838800	11.258982800	-37.809816800	C	-56.357246500	11.245252300	-37.842525100
C	-57.628434800	12.079203100	-37.266326900	C	-57.513662500	12.146755700	-37.303704600
O	-58.764868900	11.837493100	-37.683271500	O	-58.641375100	12.038981500	-37.795861700
C	-55.737819000	12.004234200	-39.006105000	C	-55.671316100	11.931931200	-39.066860400
C	-54.999209700	13.272047300	-38.560968600	C	-54.937796600	13.218936300	-38.673948000
C	-54.780671300	11.073652100	-39.768954200	C	-54.715771700	10.966370300	-39.786742100
H	-57.056373000	9.355009300	-37.426722700	H	-57.125384000	9.391829200	-37.418043800
H	-57.831265500	10.105113900	-38.648198600	H	-57.793254000	10.133338800	-38.715303500
H	-55.657844300	11.128006000	-37.009011400	H	-55.591740800	11.094698400	-37.049157100
H	-56.563397200	12.295123900	-39.697419200	H	-56.498740700	12.193141200	-39.767642100
H	-54.584821800	13.801272500	-39.442266800	H	-54.539282800	13.720125000	-39.578755300
H	-55.649912600	13.984513500	-38.020991800	H	-55.588248900	13.942940000	-38.148854300
H	-54.151376600	13.017374300	-37.891732400	H	-54.079924400	12.991616400	-38.007959900
H	-54.431242800	11.563965500	-40.702264800	H	-54.367194800	11.412326700	-40.742316400
H	-53.886603400	10.836582400	-39.154195400	H	-53.820339500	10.759442100	-39.163833500
H	-55.280759100	10.122906600	-40.029205100	H	-55.216770800	10.005272000	-40.005459500
N	-57.377385200	13.068109900	-36.348064200	N	-57.222490700	13.049342400	-36.312981300
C	-58.406875000	14.067289400	-36.036181000	C	-58.186408000	14.102496400	-35.973510500
C	-58.401324500	15.109055700	-37.175110800	C	-58.162218000	15.184180300	-37.072595600
O	-59.192366000	15.086414600	-38.121313100	O	-59.082611700	15.337355800	-37.882205900
C	-58.241517700	14.672620300	-34.632741800	C	-57.978720800	14.692003200	-34.566684300
O	-57.100845500	15.515628600	-34.472428500	O	-56.805536600	15.483256400	-34.425858800
H	-56.406994900	13.279005900	-36.065318200	H	-56.246772900	13.168106100	-35.985433200
H	-59.384323000	13.549560400	-36.099911900	H	-59.196978000	13.652265600	-36.033691200
H	-58.228130800	13.846345700	-33.891350400	H	-58.004607100	13.865922700	-33.823141300
H	-59.119847800	15.316998700	-34.424070100	H	-58.831894700	15.370415200	-34.359706900
H	-56.282533100	14.950881400	-34.497578400	H	-56.025986300	14.875608200	-34.398524100
N	-57.329797500	15.966418100	-37.132262800	N	-56.984332000	15.877755800	-37.173758000
C	-57.078019400	16.824801700	-38.263750000	C	-56.822952200	16.762466800	-38.299536500
C	-57.299000000	16.002000000	-39.542000000	C	-57.299000000	16.002000000	-39.542000000
O	-57.516943500	16.509888500	-40.627234900	O	-57.610923600	16.545928300	-40.585562000
C	-55.593896300	17.306336800	-38.284126500	C	-55.333835700	17.129053900	-38.562608100
O	-55.110741200	17.605271100	-36.960608000	O	-54.614069800	17.271874000	-37.324706700
C	-55.439013000	18.551771700	-39.144212500	C	-55.236110400	18.396879100	-39.407409000
H	-57.100441700	14.901418100	-39.429440900	H	-57.200021100	14.883511000	-39.461161900
H	-56.958514100	16.152703100	-36.180331600	H	-56.401612700	15.966680900	-36.327966400
H	-57.754292300	17.710879400	-38.294002300	H	-57.428224500	17.693808400	-38.202241400
H	-54.970839600	16.482145700	-38.704857200	H	-54.874665400	16.281083600	-39.119144800
H	-54.996953300	16.751060600	-36.494933500	H	-53.821721600	16.691969700	-37.398569700
H	-54.370788000	18.811819800	-39.282167400	H	-54.188999700	18.585333200	-39.717555300
H	-55.901262500	18.382151200	-40.136240600	H	-55.870599300	18.303315400	-40.311198300
H	-55.958196500	19.407331500	-38.665685300	H	-55.600054200	19.268306300	-38.824513300
N	-55.492000000	24.131000000	-34.333000000	N	-55.492000000	24.131000000	-34.333000000
C	-54.104947600	23.792607300	-33.991197300	C	-54.084762300	23.814450300	-34.069409100
C	-53.870091200	22.499406400	-34.789118800	C	-53.862128900	22.516608500	-34.864593800
O	-54.479152200	21.463613700	-34.478363400	O	-54.485494300	21.490828300	-34.551999000
C	-53.862738100	23.631723000	-32.475801100	C	-53.757681100	23.663453400	-32.564610300

C	-52.443922600	23.177645900	-32.071650100	C	-52.309286700	23.230736000	-32.245754800
O	-51.992954500	22.144471300	-32.696668300	O	-51.952746400	22.085800700	-32.711313100
O	-51.857708600	23.785475100	-31.153717500	O	-51.597611400	23.989122500	-31.554956700
H	-56.088701300	23.335725000	-34.062646200	H	-56.059064300	23.317821500	-34.052221200
H	-55.807106200	24.939346300	-33.780566400	H	-55.794507600	24.923783000	-33.752150400
H	-53.449229400	24.604805400	-34.366034800	H	-53.461591700	24.635260600	-34.479451800
H	-54.568466300	22.858835500	-32.097980100	H	-54.436308500	22.888917300	-32.145032700
H	-54.104965900	24.586586100	-31.966792600	H	-53.974826100	24.626020000	-32.059067300
N	-53.125584000	22.564062700	-35.929510400	N	-53.086412800	22.559546100	-35.985377200
C	-53.097282200	21.406003700	-36.830260900	C	-53.032285100	21.382626900	-36.860102000
C	-52.129887400	20.327869000	-36.302661500	C	-52.079249800	20.316122900	-36.281664400
O	-50.968029900	20.600688500	-35.981347900	O	-50.933552900	20.600337700	-35.921771200
C	-52.599193400	22.016252200	-38.162101600	C	-52.492874500	21.963696800	-38.188868900
C	-51.683419500	23.166959400	-37.709310100	C	-51.586762600	23.120695000	-37.732638200
C	-52.392201600	23.724399100	-36.461836100	C	-52.328723900	23.705684600	-36.517238100
H	-54.118206600	20.979678000	-36.906706200	H	-54.051018200	20.955358400	-36.957911400
H	-53.472769600	22.403857100	-38.726649800	H	-53.348162800	22.341511900	-38.787044100
H	-52.086363700	21.274796900	-38.805684000	H	-51.964180700	21.207339600	-38.801472600
H	-51.538169500	23.939739700	-38.489805500	H	-51.417324800	23.877328900	-38.523934000
H	-50.690857100	22.764226500	-37.429955000	H	-50.603181800	22.722666700	-37.417181300
H	-51.674910000	24.118932800	-35.714495200	H	-51.630644900	24.110828600	-35.757666300
H	-53.102902100	24.539317500	-36.725840300	H	-53.024928100	24.520227300	-36.818250400
N	-52.640897400	19.060329000	-36.262497100	N	-52.591613700	19.047027700	-36.242768800
C	-51.845174100	17.906254500	-35.841283700	C	-51.811913300	17.879120600	-35.814696700
C	-51.292000000	17.314000000	-37.146000000	C	-51.292000000	17.314000000	-37.146000000
O	-51.557952300	16.203946400	-37.573882100	O	-51.869039300	16.429810300	-37.770590600
C	-52.672538600	16.896475600	-35.028714900	C	-52.657716500	16.867134900	-35.014397800
S	-52.100757300	16.519308300	-33.320232900	S	-52.085277600	16.504877700	-33.304959000
H	-50.649941100	18.033261600	-37.722497900	H	-50.379949200	17.818718600	-37.553285900
H	-53.607472600	18.851929900	-36.569420300	H	-53.523100400	18.832512600	-36.639483400
H	-50.977020000	18.302964800	-35.275667700	H	-50.947463400	18.258970000	-35.238495300
H	-52.822539200	15.967392900	-35.608092800	H	-52.797808700	15.928131800	-35.579939500
H	-53.678179600	17.315852400	-34.830193100	H	-53.678759500	17.265821100	-34.854257800
N	-48.129000000	16.435000000	-37.652000000	N	-48.129000000	16.435000000	-37.652000000
C	-47.265329000	15.262702800	-37.482175900	C	-47.269200900	15.256475400	-37.484212400
C	-48.089145400	13.959535400	-37.322807700	C	-48.106829200	13.960187700	-37.372193800
O	-47.677046000	12.858915500	-37.684555800	O	-47.737528300	12.876146300	-37.819840400
C	-46.391139800	15.404739000	-36.229307400	C	-46.415814800	15.373256000	-36.214183900
O	-45.621363000	16.602944300	-36.347283700	O	-45.630232000	16.563168600	-36.306984300
H	-47.647184400	17.289170800	-37.337165500	H	-47.658670000	17.276263700	-37.286834000
H	-48.336030500	16.573649800	-38.648152400	H	-48.272193100	16.611793500	-38.653916700
H	-46.584699100	15.075094800	-38.345899700	H	-46.575524600	15.084128400	-38.339942000
H	-45.745354400	14.498514100	-36.163195100	H	-45.780626500	14.459244800	-36.147384800
H	-47.049455800	15.433478900	-35.331195300	H	-47.086703400	15.400259600	-35.325932100
H	-45.155002700	16.732900900	-35.502867300	H	-45.211660100	16.702704500	-35.439375500
N	-49.299809900	14.163674300	-36.708873700	N	-49.294335600	14.148068700	-36.708026300
C	-50.270978700	13.101337000	-36.558274100	C	-50.268395400	13.087327500	-36.568444800
C	-50.938294400	12.660931800	-37.904513500	C	-50.949795400	12.681324800	-37.916291100
O	-51.486560600	11.562586100	-37.998448700	O	-51.522529200	11.596582500	-38.020783400
C	-51.429322700	13.493815500	-35.632067200	C	-51.412604800	13.472909600	-35.624070300
S	-51.092434000	13.477197000	-33.820632800	S	-51.040826100	13.429399300	-33.817785500
H	-49.523698400	15.148396900	-36.505398100	H	-49.478275200	15.107115200	-36.381973200
H	-49.757733500	12.183126800	-36.201300400	H	-49.764157000	12.156479300	-36.231051800
H	-52.248079800	12.756748500	-35.752853000	H	-52.239422500	12.747308200	-35.751380500
H	-51.832385000	14.474770300	-35.941702500	H	-51.812355800	14.462648400	-35.908154200
N	-50.905980100	13.600753400	-38.891534000	N	-50.913295900	13.634198100	-38.892041800
C	-51.545884100	13.430769400	-40.189441500	C	-51.534209300	13.470007700	-40.199545800
C	-50.531000000	12.863000000	-41.188000000	C	-50.531000000	12.863000000	-41.188000000
O	-50.444300800	13.204657200	-42.350714800	O	-50.472221100	13.148649300	-42.367357900
C	-52.186957000	14.719037700	-40.701553400	C	-52.144797900	14.763039500	-40.735549600
H	-49.846736300	12.081084700	-40.741936600	H	-49.833133900	12.106564200	-40.719770000
H	-50.534433300	14.526102400	-38.654943500	H	-50.454072300	14.523078000	-38.674512400
H	-52.309019600	12.626515500	-40.057848900	H	-52.315707200	12.683600400	-40.069091400
H	-52.659192100	14.538602200	-41.685215900	H	-52.584372500	14.580416000	-41.733777900
H	-52.948405500	15.090632200	-39.991829600	H	-52.923715900	15.146389700	-40.052284200
H	-51.423758700	15.511254800	-40.844193200	H	-51.370391100	15.548238500	-40.854150200
Fe	-52.938043400	14.394849600	-32.608625700	Fe	-52.862635600	14.383769000	-32.577669900

C	-54.071139000	14.242546200	-34.073726100
N	-54.791074600	14.122415700	-35.009369200
C	-54.196744600	15.358457300	-31.604941800
N	-54.905426500	16.042763100	-30.944287700
C	-53.514507500	12.838434600	-32.084429900
O	-53.945259700	11.795003400	-31.799976300
Ni	-50.157157500	15.355597100	-32.993055400

CS_BP86

N	-48.065000000	22.088000000	-34.966000000
C	-47.559756200	20.720399600	-34.769117400
C	-47.141000000	20.185000000	-36.148000000
O	-47.654062900	19.248714300	-36.728975500
C	-48.507169000	19.729760700	-34.090653000
C	-47.806898300	18.382484100	-33.823459200
C	-48.785745300	17.283696300	-33.549442900
O	-49.226447500	16.523276100	-34.476650300
O	-49.203068700	17.061603300	-32.355250000
H	-49.045055400	22.021227900	-35.282281800
H	-48.089645000	22.579885500	-34.064715900
H	-46.335172500	20.806797100	-36.642951100
H	-46.606482600	20.802825800	-34.191432500
H	-49.374953100	19.595485800	-34.766594300
H	-48.899655900	20.162018900	-33.149669800
H	-47.126890400	18.464770800	-32.951810700
H	-47.218027800	18.070753100	-34.706531800
N	-46.173000000	11.189000000	-33.543000000
C	-46.299860400	12.477765900	-32.880056800
C	-45.849590100	12.277405100	-31.398625900
O	-46.256434100	11.276588900	-30.794032000
C	-47.794755500	12.838370900	-33.010486300
S	-48.113383000	14.533314700	-32.448069700
H	-45.183540800	10.945512800	-33.681472200
H	-46.557066900	10.470597700	-32.911346700
H	-45.684000000	13.240000000	-33.434000000
H	-48.385960900	12.094723900	-32.435126300
H	-48.059634900	12.738719700	-34.079566900
N	-44.870000800	13.073172900	-30.843327100
C	-44.777323300	14.528427300	-30.933603200
C	-45.401062900	15.102125900	-29.636524200
O	-44.844393000	14.939717300	-28.548076900
H	-44.578724900	12.723919500	-29.920102700
H	-45.298356600	14.878399800	-31.846480400
H	-43.714612200	14.845947300	-30.976507000
N	-46.620235700	15.681163300	-29.814037600
C	-47.498483500	16.151656600	-28.750298200
C	-48.517569300	15.085789200	-28.313678600
O	-49.050321300	15.104630500	-27.198993800
C	-48.256000000	17.430000000	-29.226000000
C	-47.232000000	18.397000000	-29.828000000
C	-49.003000000	18.127000000	-28.108000000
C	-47.851000000	19.602000000	-30.514000000
H	-47.012486400	15.657214300	-30.766796900
H	-46.868641100	16.376852300	-27.865688700
H	-48.962650400	17.123629300	-30.025887000
H	-46.607624600	17.860094900	-30.569593700
H	-46.525458500	18.718469500	-29.029008100
H	-49.592169700	18.979748700	-28.500314900
H	-49.695953300	17.448472700	-27.572873300
H	-48.298705600	18.533324200	-27.351079200
H	-47.100137600	20.161082500	-31.109858600
H	-48.672645000	19.295242700	-31.189688500
H	-48.276470800	20.330267000	-29.792299000
N	-48.808120500	14.153802600	-29.268869900
C	-49.887396500	13.213516000	-29.112001700
C	-49.455779100	11.757470700	-28.825981500
O	-50.244144100	10.845090100	-29.152400500
C	-50.787020400	13.158242300	-30.373045400

C	-53.970065000	14.089556000	-34.048132600
N	-54.653304500	13.805906100	-34.974412300
C	-54.195629300	15.323756700	-31.641626800
N	-54.945308600	15.994976400	-31.015099500
C	-53.365450700	12.821482000	-31.991990000
O	-53.747837600	11.770316200	-31.672501800
Ni	-50.164299800	15.336609800	-32.985439600

CS_PBE0

N	-48.065000000	22.088000000	-34.966000000
C	-47.595953300	20.709437100	-34.788703900
C	-47.141000000	20.185000000	-36.148000000
O	-47.632280000	19.263468900	-36.737731700
C	-48.569015600	19.731292600	-34.149006200
C	-47.877231000	18.392470200	-33.870345100
C	-48.824550700	17.284799400	-33.578461300
O	-49.255266900	16.506925300	-34.479995700
O	-49.214405800	17.044381000	-32.398133600
H	-49.013144100	22.064407900	-35.330637400
H	-48.111099700	22.550624700	-34.067174500
H	-46.322137800	20.779425700	-36.604331500
H	-46.675418300	20.766881200	-34.187697100
H	-49.407173900	19.604037900	-34.834705300
H	-48.971094500	20.156059900	-33.227613800
H	-47.209075600	18.488767400	-33.011061400
H	-47.295939300	18.083228900	-34.738404500
N	-46.173000000	11.189000000	-33.543000000
C	-46.328291100	12.492665000	-32.926235900
C	-45.987371500	12.320379000	-31.432658000
O	-46.482339300	11.381054100	-30.831788500
C	-47.790722800	12.874729000	-33.152286000
S	-48.088487000	14.534568500	-32.546837500
H	-45.196196500	10.942505300	-33.644218200
H	-46.602653100	10.493133800	-32.943278400
H	-45.684000000	13.240000000	-33.434000000
H	-48.421594700	12.136014100	-32.651406000
H	-47.974034400	12.819917100	-34.224375100
N	-45.007332900	13.065812900	-30.842941600
C	-44.847904400	14.503536000	-30.936760100
C	-45.460588800	15.090902100	-29.660658500
O	-44.912981800	14.919223000	-28.586401100
H	-44.785700200	12.716508200	-29.917748100
H	-45.331267600	14.870174200	-31.842952500
H	-43.788289500	14.767697900	-30.958976100
N	-46.649266600	15.693668800	-29.829474200
C	-47.514035800	16.158598100	-28.767050500
C	-48.539321800	15.106443500	-28.363480800
O	-49.091738600	15.126246200	-27.277708200
C	-48.256000000	17.430000000	-29.226000000
C	-47.232000000	18.397000000	-29.828000000
C	-49.003000000	18.127000000	-28.108000000
C	-47.851000000	19.602000000	-30.514000000
H	-47.017905300	15.718289900	-30.771161600
H	-46.896054800	16.366959400	-27.891293500
H	-48.958377000	17.142504000	-30.015249000
H	-46.614716500	17.874178800	-30.559953000
H	-46.545625200	18.719969600	-29.035614400
H	-49.549346700	18.987164600	-28.498731700
H	-49.712348500	17.467060600	-27.607446400
H	-48.306980200	18.495362800	-27.348553900
H	-47.102623500	20.147590600	-31.094650800
H	-48.653985600	19.296821000	-31.185919300
H	-48.273132500	20.314378500	-29.801556900
N	-48.804445900	14.186763900	-29.313204500
C	-49.884949100	13.261079200	-29.210271400
C	-49.499128200	11.819323300	-28.849235100
O	-50.297640100	10.932812200	-29.151410100
C	-50.654917600	13.176994400	-30.538574500

S	-51.279793200	14.802348500	-31.031307400	S	-51.190666400	14.784162700	-31.186957800
H	-48.399996300	14.231254400	-30.221335200	H	-48.360452100	14.257717100	-30.224829500
H	-50.482237400	13.554114100	-28.233532400	H	-50.551446400	13.617326700	-28.416491300
H	-51.715408400	12.611043000	-30.132114400	H	-51.547760800	12.575436200	-30.392315400
H	-50.287551100	12.600626400	-31.190344900	H	-50.054252100	12.680387400	-31.301734300
N	-48.271498800	11.448679800	-28.232606600	N	-48.346520100	11.506623000	-28.234325700
C	-47.918647900	10.011918800	-28.174308500	C	-48.002941300	10.085560700	-28.168509200
C	-48.911220200	9.202848500	-27.316221500	C	-48.947453200	9.273810400	-27.295219700
O	-49.184164100	8.029682100	-27.575822000	O	-49.161659200	8.099503400	-27.529777600
C	-46.471193000	10.019087100	-27.634272000	C	-46.567434600	10.082484300	-27.637898300
C	-46.395977100	11.306708700	-26.797645600	C	-46.476848100	11.370449400	-26.829722500
C	-47.235652000	12.305389100	-27.611093700	C	-47.305767500	12.353801800	-27.643980300
H	-47.978135600	9.571978100	-29.187434000	H	-48.070127800	9.644964800	-29.163029400
H	-45.785114000	10.086590700	-28.501540900	H	-45.885404600	10.117862500	-28.488880900
H	-46.233987100	9.097825600	-27.068196600	H	-46.351693800	9.183674200	-27.060078900
H	-45.362640200	11.674947300	-26.645389100	H	-45.454825900	11.726427800	-26.696080800
H	-46.839992900	11.148042100	-25.792051400	H	-46.909992800	11.232440600	-25.835145100
H	-47.694648600	13.098388800	-26.990876200	H	-47.741407100	13.144055200	-27.034623500
H	-46.616626400	12.789334400	-28.390939000	H	-46.696135900	12.808150800	-28.424274100
N	-49.440170000	9.868867600	-26.242009000	N	-49.488830000	9.920677800	-26.240348700
C	-50.454395600	9.275189000	-25.382340500	C	-50.448093100	9.289143700	-25.366841000
C	-51.767639800	8.921193800	-26.135691800	C	-51.743209400	8.889078000	-26.090126900
O	-52.458134500	7.967607200	-25.777816700	O	-52.374118300	7.916169800	-25.728715100
C	-50.730000000	10.208000000	-24.166000000	C	-50.730000000	10.208000000	-24.166000000
C	-51.957000000	9.716000000	-23.400000000	C	-51.957000000	9.716000000	-23.400000000
C	-49.514000000	10.339000000	-23.256000000	C	-49.514000000	10.339000000	-23.256000000
H	-49.253060300	10.870228800	-26.155112000	H	-49.348057800	10.914307700	-26.163640600
H	-50.084840600	8.293542500	-25.017056500	H	-50.034452800	8.341002100	-25.012018000
H	-50.971876600	11.213653500	-24.586135000	H	-50.970755300	11.201206200	-24.571542200
H	-52.099600100	10.317456100	-22.477935500	H	-52.096009200	10.319786700	-22.499309300
H	-52.884888100	9.789503100	-23.995825100	H	-52.865554100	9.784636600	-23.996243200
H	-51.845168200	8.653675500	-23.102541900	H	-51.839448100	8.672830900	-23.099203900
H	-49.708912200	11.064899300	-22.440409500	H	-49.711833500	11.058937800	-22.458928400
H	-49.265722800	9.363839500	-22.785771700	H	-49.278722800	9.377735100	-22.790011300
H	-48.609579100	10.683213400	-23.798287500	H	-48.624867700	10.673353100	-23.795913800
N	-52.130068700	9.779176200	-27.140083300	N	-52.148991600	9.709714400	-27.084635600
C	-53.357907600	9.578245300	-27.893062400	C	-53.368256100	9.461515600	-27.811375000
C	-53.280209600	8.449898600	-28.952182100	C	-53.257392900	8.388952400	-28.901457800
O	-54.305282600	7.952513100	-29.418171100	O	-54.262405200	7.868785100	-29.347636400
C	-53.787773600	10.843234600	-28.638261800	C	-53.887561500	10.724373000	-28.468350800
O	-53.943446200	11.940139700	-27.745769400	O	-54.155202900	11.706232000	-27.486975800
H	-51.439132700	10.432216900	-27.525872400	H	-51.513714800	10.403575200	-27.448742200
H	-54.149487200	9.266084200	-27.182654000	H	-54.112882900	9.085529600	-27.107523700
H	-54.729101100	10.588251200	-29.171010000	H	-54.787862300	10.448890400	-29.024349700
H	-53.019325400	11.058426600	-29.418831000	H	-53.139860600	11.086250300	-29.187100100
H	-54.461243400	12.609680800	-28.230973100	H	-54.737045000	12.357472400	-27.886921900
N	-52.016543900	8.127739800	-29.368413100	N	-52.010358500	8.120781400	-29.339011200
C	-51.784820500	6.954919700	-30.191374700	C	-51.745818300	6.965801800	-30.158563200
C	-51.667000000	5.759000000	-29.241000000	C	-51.667000000	5.759000000	-29.241000000
O	-50.957034900	4.790775000	-29.424937600	O	-51.056679100	4.756239700	-29.486563200
C	-50.567432900	7.070593900	-31.126856900	C	-50.492535700	7.108879900	-31.015334600
C	-50.697519200	8.082680300	-32.229682700	C	-50.631213600	8.033632800	-32.174872800
N	-50.796378500	7.713189700	-33.559412000	N	-50.807658100	7.560291100	-33.451248100
C	-50.732019700	9.471669400	-32.120616700	C	-50.611240800	9.401837000	-32.187880000
C	-50.886036200	8.844161200	-34.245760900	C	-50.890301700	8.616377300	-34.218072500
N	-50.853362300	9.938128000	-33.414575200	N	-50.779061800	9.757985500	-33.499410900
H	-51.215550800	8.489902500	-28.839003300	H	-51.230759500	8.503816600	-28.824850600
H	-52.722760100	6.797372600	-30.773536700	H	-52.625997900	6.810548000	-30.793711800
H	-52.310821700	5.857365100	-28.316836300	H	-52.236791700	5.873368600	-28.295473700
H	-50.404583200	6.074224400	-31.580258100	H	-50.244380400	6.115873700	-31.392076200
H	-49.675777800	7.277857700	-30.495805900	H	-49.666834800	7.416108700	-30.365373800
H	-50.688624000	10.130467400	-31.247441000	H	-50.513014200	10.118621700	-31.389298200
H	-50.893635900	10.927078900	-33.683867300	H	-50.792427700	10.696339500	-33.861005600
H	-50.966896500	8.946812900	-35.336190400	H	-51.019312200	8.620191300	-35.290357200
N	-58.620000000	12.567000000	-28.890000000	N	-58.620000000	12.567000000	-28.890000000
C	-57.506568400	13.453038100	-28.604388700	C	-57.554613100	13.476501500	-28.564182200
C	-57.954174200	14.932434800	-28.470182800	C	-58.041181600	14.925129600	-28.432879200

O	-59.118038400	15.267822500	-28.756421300
C	-56.448331600	13.269511700	-29.702488300
H	-59.423662900	12.841674500	-28.309397100
H	-58.927398600	12.739951400	-29.858841800
H	-57.053973300	13.153718900	-27.627259000
H	-55.516149600	13.835934900	-29.516311300
H	-56.226225800	12.189627000	-29.805959200
H	-56.844000600	13.625541000	-30.674367500
N	-57.058632500	15.864606500	-28.021579100
C	-57.504606800	17.256022000	-27.797999700
C	-58.126675500	17.967274200	-29.015108400
O	-58.935480100	18.878857500	-28.840093300
C	-56.233106500	17.955622700	-27.265554600
C	-55.476551700	16.822354200	-26.554413000
C	-55.702881400	15.625286100	-27.491360900
H	-58.314441300	17.269395200	-27.036565100
H	-56.481125900	18.814670700	-26.614300300
H	-55.629287300	18.338720500	-28.115118300
H	-55.928851700	16.620742800	-25.560302000
H	-54.401309800	17.039115400	-26.398418500
H	-54.962925500	15.626143900	-28.321795800
H	-55.643805700	14.649864800	-26.967799900
N	-57.696783500	17.548306700	-30.245878300
C	-58.207599200	18.135107400	-31.475728900
C	-59.338201900	17.311616500	-32.140281000
O	-59.753770400	17.606985300	-33.265691700
C	-57.079813000	18.374588000	-32.494932200
C	-55.961056200	19.290222900	-31.976798900
C	-54.642726800	19.000755100	-32.707019000
N	-53.496391800	19.777304400	-32.248859200
C	-52.703279100	19.474311800	-31.214163600
N	-51.589600200	20.214936300	-31.014820200
N	-53.000300100	18.449903600	-30.372108500
H	-56.932671500	16.859259300	-30.315204800
H	-58.678315500	19.099464000	-31.181043800
H	-57.543208100	18.778403100	-33.415984900
H	-56.666459500	17.382109300	-32.777666600
H	-55.814125100	19.122159700	-30.888828500
H	-56.229381500	20.359705800	-32.104466400
H	-54.755125600	19.227025400	-33.783142200
H	-54.407847900	17.920794900	-32.606293600
H	-53.264439400	20.657532500	-32.753251200
H	-51.225713100	20.261718500	-30.062960400
H	-51.521965300	21.098955100	-31.619964500
H	-52.223287500	18.052845100	-29.843121100
H	-53.740835400	17.766209500	-30.607442800
N	-59.847056400	16.272812100	-31.407941000
C	-60.874846900	15.443524500	-31.969532500
C	-60.497000000	13.991000000	-32.170000000
O	-59.460900000	13.469260100	-31.801586800
H	-59.479337800	16.040025700	-30.469822000
H	-61.160451600	15.884323000	-32.953427400
H	-61.812627400	15.440528600	-31.358864700
H	-61.305932500	13.381414600	-32.678951000
N	-56.890000000	9.950000000	-38.249000000
C	-56.774405600	10.326731400	-36.832912900
C	-57.814174500	11.457526800	-36.634482800
O	-58.995181900	11.194491500	-36.880957900
C	-55.278861200	10.508976600	-36.468989700
C	-54.549405200	11.559884600	-37.324189700
C	-55.040848800	10.668052600	-34.960250600
H	-56.506094200	9.005431500	-38.383916300
H	-57.893217100	9.895878600	-38.475827700
H	-57.166141900	9.533487200	-36.142265400
H	-54.836299300	9.524524700	-36.750187500
H	-53.459535400	11.357032500	-37.336444200
H	-54.921661400	11.507221100	-38.367226300

O	-59.195184500	15.226212500	-28.719127600
C	-56.471275000	13.334962200	-29.623286400
H	-59.439466600	12.778069100	-28.333332900
H	-58.895963500	12.708205000	-29.855603900
H	-57.141773200	13.186376000	-27.586488300
H	-55.584482000	13.933169700	-29.420041500
H	-56.201666200	12.280379500	-29.709308700
H	-56.856354500	13.654659400	-30.594212500
N	-57.181373200	15.871821000	-27.995375600
C	-57.671430400	17.230255400	-27.774752300
C	-58.247598200	17.938473400	-28.994218800
O	-59.045373200	18.842841600	-28.838073300
C	-56.453657000	17.959660700	-27.202508300
C	-55.687090800	16.852896600	-26.490605800
C	-55.846614900	15.671310100	-27.437189300
H	-58.494009200	17.211873200	-27.053527200
H	-56.748360800	18.784234400	-26.555241800
H	-55.849193300	18.370150700	-28.016822200
H	-56.153443700	16.628179500	-25.527018400
H	-54.640095100	17.099997200	-26.310166100
H	-55.093615200	15.703844200	-28.230945900
H	-55.773913400	14.710233900	-26.925192000
N	-57.792346900	17.531514800	-30.198011800
C	-58.249267300	18.113640300	-31.436064500
C	-59.379920800	17.328157700	-32.110810800
O	-59.807872300	17.680231400	-33.196059500
C	-57.091748300	18.270849300	-32.416099000
C	-55.991297600	19.193595800	-31.911385800
C	-54.701303300	18.925820200	-32.670306700
N	-53.566327400	19.721212600	-32.251500900
C	-52.776722300	19.459747900	-31.227166800
N	-51.679003300	20.199218900	-31.048995600
N	-53.054030200	18.464239800	-30.368415400
H	-57.062891600	16.831350700	-30.239467500
H	-58.675890700	19.090980700	-31.190347200
H	-57.499625000	18.629148300	-33.362224100
H	-56.678869000	17.274791600	-32.613476400
H	-55.825288600	19.028696900	-30.842648700
H	-56.277626400	20.241895900	-32.034592600
H	-54.850829800	19.146953600	-33.726358100
H	-54.450672600	17.865861800	-32.579180200
H	-53.355337000	20.572956500	-32.775924100
H	-51.336455500	20.285021900	-30.107570900
H	-51.612450300	21.054810400	-31.658635800
H	-52.286118500	18.115654100	-29.821418200
H	-53.764666300	17.772185100	-30.589359600
N	-59.853947400	16.257316500	-31.438876000
C	-60.868549900	15.436216300	-32.006356700
C	-60.497000000	13.991000000	-32.170000000
O	-59.481700100	13.482100200	-31.782135600
H	-59.494540500	16.020754100	-30.520922400
H	-61.132040400	15.847419900	-32.987698400
H	-61.798536300	15.448714100	-31.415944000
H	-61.280552600	13.383982100	-32.674061600
N	-56.890000000	9.950000000	-38.249000000
C	-56.799017400	10.272989500	-36.829293700
C	-57.898356900	11.313748300	-36.603869600
O	-59.056272900	10.941367000	-36.684132000
C	-55.334073900	10.536670600	-36.461894700
C	-54.644258100	11.625686400	-37.280243400
C	-55.131092700	10.705689300	-34.963568700
H	-56.369999500	9.101602300	-38.440884900
H	-57.859799600	9.770627300	-38.483005500
H	-57.127979900	9.438017300	-36.190091000
H	-54.843263900	9.592535300	-36.737233000
H	-53.569019500	11.435965600	-37.319100300
H	-55.024007900	11.627820400	-38.303853600

H	-54.688285700	12.595139300	-36.947719900	H	-54.771376200	12.623444300	-36.849997600
H	-53.957941800	10.619241000	-34.726588700	H	-54.065482800	10.719321800	-34.721336900
H	-55.418520900	11.634909200	-34.572759600	H	-55.563945100	11.638303500	-34.595622400
H	-55.535260100	9.860366800	-34.382115200	H	-55.581275100	9.882396300	-34.403359700
N	-57.418004900	12.696270400	-36.195530800	N	-57.567085100	12.603775600	-36.362722100
C	-58.396795700	13.779332700	-36.072039800	C	-58.592832800	13.625194800	-36.276406600
C	-58.256687400	14.743562400	-37.266801300	C	-58.394129400	14.657513200	-37.379938300
O	-58.919575900	14.620170200	-38.302012500	O	-59.083550900	14.678693800	-38.383126900
C	-58.374268400	14.459334200	-34.690691400	C	-58.702872800	14.216259600	-34.876339600
O	-57.276371500	15.332646500	-34.442438700	O	-57.608573500	15.003617400	-34.464096000
H	-56.423613600	12.920564900	-36.082092700	H	-56.598638400	12.880326800	-36.372548100
H	-59.378928100	13.282210200	-36.203181100	H	-59.532079500	13.117131500	-36.498818000
H	-58.425821300	13.659754200	-33.923023000	H	-58.867585000	13.385189200	-34.185836300
H	-59.279594700	15.094076700	-34.595327800	H	-59.572152000	14.879162100	-34.843868400
H	-56.425456300	14.821965100	-34.463394400	H	-56.812100700	14.463748300	-34.367249500
N	-57.275632800	15.686284300	-37.123746400	N	-57.351278300	15.497881900	-37.186428600
C	-57.035766200	16.646810900	-38.178257600	C	-57.059750400	16.527870300	-38.144060600
C	-57.299000000	16.002000000	-39.542000000	C	-57.299000000	16.002000000	-39.542000000
O	-57.702326800	16.629240400	-40.505657100	O	-57.627638200	16.699302400	-40.462871100
C	-55.537989700	17.086879900	-38.177653100	C	-55.564448500	16.905095600	-38.073297100
O	-55.030145500	17.259207400	-36.847563600	O	-55.122491000	17.054486900	-36.736724300
C	-55.354830100	18.396928200	-38.932784600	C	-55.299652400	18.205654400	-38.795635800
H	-56.899646100	14.951163100	-39.635659800	H	-57.007199300	14.943679500	-39.689053900
H	-57.013645800	15.899427100	-36.139784900	H	-57.062477600	15.619995400	-36.220001500
H	-57.687686200	17.546010000	-38.084355600	H	-57.673808500	17.423382700	-37.990674000
H	-54.951132300	16.280725500	-38.680742100	H	-54.992600200	16.094112500	-38.547711600
H	-54.960915200	16.369522000	-36.431414100	H	-55.054042700	16.181485000	-36.314413700
H	-54.280460800	18.633854200	-39.065336000	H	-54.226157800	18.391828600	-38.866731100
H	-55.833032100	18.337542200	-39.929698800	H	-55.718110900	18.184900200	-39.802050900
H	-55.838721400	19.223779900	-38.372838800	H	-55.768216600	19.030272300	-38.251740400
N	-55.492000000	24.131000000	-34.333000000	N	-55.492000000	24.131000000	-34.333000000
C	-54.106251100	23.762011200	-34.001551500	C	-54.139970200	23.683835500	-34.005210700
C	-53.859685700	22.458050500	-34.783892200	C	-53.947161900	22.383443700	-34.778664200
O	-54.377304000	21.383611100	-34.457720300	O	-54.486501700	21.338955000	-34.446913100
C	-54.004786400	23.580591400	-32.484599300	C	-54.039165700	23.495189000	-32.505260000
C	-52.635471200	23.111511000	-31.960629500	C	-52.681563400	23.006431200	-32.000473900
O	-52.036433400	22.217873500	-32.678303200	O	-52.087318500	22.171713200	-32.766141900
O	-52.227499900	23.541166900	-30.863884200	O	-52.294387700	23.375406100	-30.893877900
H	-55.746674000	24.985365100	-33.819015800	H	-55.740354900	24.920789200	-33.749955000
H	-55.574295700	24.367353500	-35.331451700	H	-55.559767100	24.434860500	-35.297207100
H	-53.347632500	24.524129300	-34.306469700	H	-53.365544800	24.400539900	-34.308216600
H	-54.757721000	22.818102500	-32.188491000	H	-54.793517100	22.762267900	-32.199760700
H	-54.282723500	24.526825000	-31.976394500	H	-54.282224300	24.435604100	-32.004184000
N	-53.187714300	22.562114400	-35.976546400	N	-53.262919300	22.455989600	-35.946189300
C	-53.133123200	21.389884600	-36.855455800	C	-53.229699300	21.299596500	-36.817962700
C	-52.154843900	20.331687900	-36.306819800	C	-52.211808200	20.265060000	-36.342522500
O	-50.996148200	20.622386200	-35.988926500	O	-51.048388100	20.557663700	-36.123842000
C	-52.632217800	21.985457300	-38.192330400	C	-52.810868900	21.896394500	-38.164363100
C	-51.722733100	23.144648900	-37.748512400	C	-51.913819300	23.066306300	-37.772995600
C	-52.431840500	23.710896500	-36.503503900	C	-52.544738900	23.602445000	-36.490479100
H	-54.145597800	20.944565500	-36.936723400	H	-54.222453800	20.846173900	-36.850723300
H	-53.504905300	22.361441200	-38.766436100	H	-53.706001000	22.248244200	-38.683931300
H	-52.113575700	21.238625600	-38.824951400	H	-52.312166900	21.171968300	-38.809748600
H	-51.578317800	23.910559400	-38.536077800	H	-51.851007200	23.829302800	-38.550200400
H	-50.729499300	22.747547300	-37.464144200	H	-50.907552500	22.702743100	-37.564805000
H	-51.712785200	24.091979200	-35.751277200	H	-51.791691700	23.962219400	-35.787261400
H	-53.117903500	24.546459300	-36.773348000	H	-53.235001200	24.427068200	-36.703751400
N	-52.642423200	19.054719500	-36.256945700	N	-52.678321300	19.000430500	-36.237244700
C	-51.823799400	17.918013400	-35.838547400	C	-51.831537200	17.891725600	-35.843632700
C	-51.292000000	17.314000000	-37.146000000	C	-51.292000000	17.314000000	-37.146000000
O	-51.506567300	16.178715300	-37.537143800	O	-51.468990700	16.191103300	-37.538485400
C	-52.620012200	16.905691200	-35.005861400	C	-52.591061600	16.867512200	-35.014312100
S	-52.075689200	16.580168500	-33.285029200	S	-52.054545300	16.542404400	-33.320623200
H	-50.714468100	18.051098000	-37.767898300	H	-50.751503300	18.059353400	-37.757537700
H	-53.613734300	18.828920000	-36.520201000	H	-53.650205400	18.788842200	-36.421507500
H	-50.946407700	18.333319200	-35.300729500	H	-50.978826700	18.317541900	-35.312516300
H	-52.721981200	15.959452200	-35.563656300	H	-52.694290400	15.939815700	-35.565256600

H	-53.649096000	17.279814900	-34.847391000	H	-53.610304200	17.218983500	-34.845837300
N	-48.129000000	16.435000000	-37.652000000	N	-48.129000000	16.435000000	-37.652000000
C	-47.249433500	15.273685400	-37.526474200	C	-47.251560500	15.280369700	-37.568611900
C	-48.054685400	13.963251800	-37.349185900	C	-48.025875000	13.988289400	-37.302768500
O	-47.629386300	12.863281100	-37.699889800	O	-47.561572400	12.893081300	-37.552763300
C	-46.342490100	15.417898800	-36.296731900	C	-46.253854600	15.437415000	-36.435435800
O	-45.594322900	16.627261500	-36.426684300	O	-45.447607700	16.569562700	-36.707721400
H	-47.633884300	17.298320300	-37.386968700	H	-47.635528800	17.285953900	-37.408557600
H	-48.423559200	16.551938300	-38.628508200	H	-48.467099400	16.553051000	-38.598439500
H	-46.590506300	15.100802700	-38.410623400	H	-46.676894900	15.097133400	-38.485595400
H	-45.682542100	14.520436400	-36.254556300	H	-45.655029400	14.521375200	-36.383468600
H	-46.978585200	15.432130500	-35.381423300	H	-46.800168000	15.560930000	-35.490717500
H	-45.103342700	16.757515200	-35.596393200	H	-44.795433200	16.652847800	-36.009295400
N	-49.259524800	14.154540800	-36.723709900	N	-49.241095100	14.181535700	-36.744147700
C	-50.166102200	13.055245100	-36.466309200	C	-50.136541100	13.092631300	-36.481411200
C	-50.740876500	12.398153300	-37.758935600	C	-50.723555500	12.437023300	-37.748699700
O	-51.033696800	11.202530700	-37.771789900	O	-51.072404400	11.273814700	-37.717852700
C	-51.368369600	13.493745400	-35.616213700	C	-51.321976100	13.543743200	-35.635329300
S	-51.089881000	13.531691900	-33.795947100	S	-51.071357500	13.586320500	-33.843441000
H	-49.499796800	15.137784400	-36.529398100	H	-49.512466100	15.148981300	-36.618758900
H	-49.615575900	12.220162400	-35.982077000	H	-49.591404300	12.277917000	-35.996857100
H	-52.192024400	12.758769500	-35.724997300	H	-52.134079500	12.816069900	-35.729843900
H	-51.756669200	14.461639400	-35.983868000	H	-51.706483600	14.488464300	-36.010708000
N	-50.950838200	13.252164800	-38.805523100	N	-50.864806800	13.247670700	-38.818436200
C	-51.562665300	12.814584500	-40.054330200	C	-51.521209100	12.827685800	-40.036559600
C	-50.531000000	12.863000000	-41.188000000	C	-50.531000000	12.863000000	-41.188000000
O	-50.776461700	13.180531800	-42.334595700	O	-50.796576300	13.211705000	-42.303844200
C	-52.858221700	13.551011800	-40.396790200	C	-52.789459600	13.605833500	-40.324633200
H	-49.490151500	12.562215600	-40.858036900	H	-49.509418700	12.524156100	-40.911349000
H	-50.772283500	14.252298700	-38.657886100	H	-50.667343200	14.229618000	-38.690659600
H	-51.764671400	11.727073300	-39.893847800	H	-51.753455200	11.764541400	-39.883112900
H	-53.224012700	13.223890500	-41.388598400	H	-53.220012400	13.282855600	-41.272352700
H	-53.637863600	13.346633000	-39.639122100	H	-53.517941200	13.447275200	-39.529276500
H	-52.690135400	14.646043900	-40.449940200	H	-52.580006900	14.675288500	-40.403234100
Fe	-52.928505500	14.454368300	-32.598783500	Fe	-52.910988600	14.458757500	-32.673460500
C	-54.064392100	14.399669400	-34.070057700	C	-54.129038800	14.438110700	-34.116479200
N	-54.760865700	14.394130200	-35.031890100	N	-54.826129200	14.487583200	-35.045502000
C	-54.160827900	15.409043800	-31.554346600	C	-54.131952500	15.373179500	-31.547848700
N	-54.855533800	16.078061200	-30.864614800	N	-54.804112000	15.999560400	-30.836872700
C	-53.528192400	12.904698900	-32.074408900	C	-53.529226300	12.893460900	-32.122395200
O	-53.963077400	11.872995300	-31.757069300	O	-53.965522900	11.903284800	-31.778243900
Ni	-50.139486700	15.401243200	-32.985266400	Ni	-50.108526100	15.418754700	-33.055961400
